# Photoinduced single-crystal-to-single-crystal phase transition and photosalient effect of a gold(i) isocyanide complex with shortening of intermolecular aurophilic bonds†
†Electronic supplementary information (ESI) available: X-ray crystallographic data, optical properties, DFT calculations, and other additional information. CCDC [987280 and 987281]. For ESI and crystallographic data in CIF or other electronic format see DOI: 10.1039/c4sc02676d



**DOI:** 10.1039/c4sc02676d

**Published:** 2014-12-15

**Authors:** Tomohiro Seki, Kenta Sakurada, Mai Muromoto, Hajime Ito

**Affiliations:** a Division of Chemical Process Engineering and Frontier Chemistry Center (FCC) , Graduate School of Engineering , Hokkaido University , Sapporo , Hokkaido 060-8628 , Japan . Email: hajito@eng.hokudai.ac.jp

## Abstract

We report the first photoinduced single-crystal-to-single-crystal (SCSC) phase transition of a gold complex that involves the shortening of intermolecular aurophilic bonds. This is also the first solid state photochromism of a gold complex. Upon UV irradiation, the blue-emitting crystals of the gold(i) isocyanide complex **1** (**1B**) transform into the weakly yellow-emitting polymorph **1Y**. X-ray diffraction analyses reveal that this phase transition proceeds in an SCSC manner. After phase transition from **1B** to **1Y**, the intermolecular Au···Au separation decreases from 3.5041(14) to 3.2955(6) Å, resulting in a red-shifted emission. The photoinduced shortening of the aurophilic bond in the excited state initiates the change in the crystal structure from **1B** to **1Y**. Moreover, the crystal **1B** showed a photosalient effect: the **1B** crystals jump upon irradiation with strong UV light owing to the phase transition into **1Y**. The aurophilic bond formation in the crystal generates the mechanical movement of the crystal.

## Abstract 2

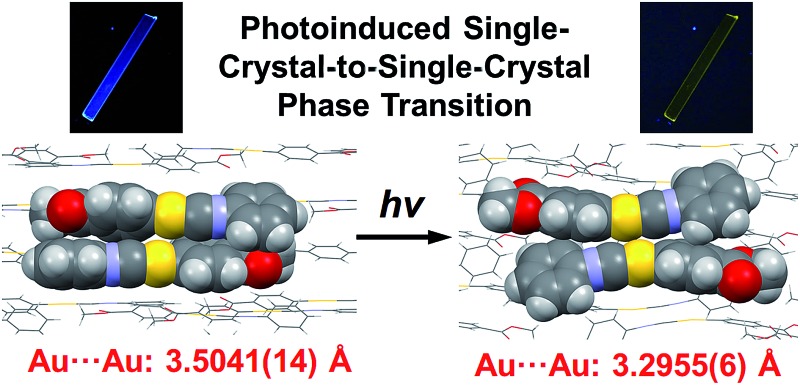
We report the first photoinduced single-crystal-to-single-crystal phase transition of a gold complex that involves shortening of intermolecular aurophilic bonds. The gold(i) isocyanide complex also shows a photosalient effect.

## Introduction

Photochromic organic solid materials attract considerable attention because of their wide applicability in sensing and recording devices.^1^ Changes in the solid structures of these materials are generally based on photochromic components exhibiting intramolecular photoinduced configurational alterations (*e.g.*, stilbene^2^ and azobenzene^3^) and intramolecular covalent bond formation/cleavage (*e.g.*, diarylethene^4^ and spiropyran^5^). Moreover, some organometallic complexes show solid-state photoinduced molecular structural alteration through the isomerization of their ligands.^6^ However, photochromic materials whose properties are based on changes in intermolecular noncovalent bonding interactions without chemical transformation or configurational alternation upon photoirradiation remain rare.

The luminescence properties of many gold complexes are sensitive to their molecular arrangements and intermolecular interaction patterns.^7^ In particular, aurophilic interactions, which are noncovalent interactions between Au atoms, strongly affect the emission properties of gold complexes in both the solid state and solution.^8^ Gold complexes are currently considered to be an important research field because of their unique structural and luminescence properties and responses to stimuli.^9^ Single-crystal-to-single-crystal (SCSC) phase transitions of gold complexes can be induced by heating,^10^ pressure,^11^ and contact with a solvent.^12^ We recently reported that mechanical stimulation or solid seeding can also induce SCSC phase transitions of gold(i) isocyanide complexes.^13^ Theoretically, photoirradiation can change the aurophilic bonding structure.^14^ Indeed, transient shortening and rapid relaxation of aurophilic bonding in solution has been reported.^15^ However, solid-state phase changes of gold complexes where the photoinduced change of aurophilic interactions is a dominant factor have not been reported.^16^

**Fig. 1:**
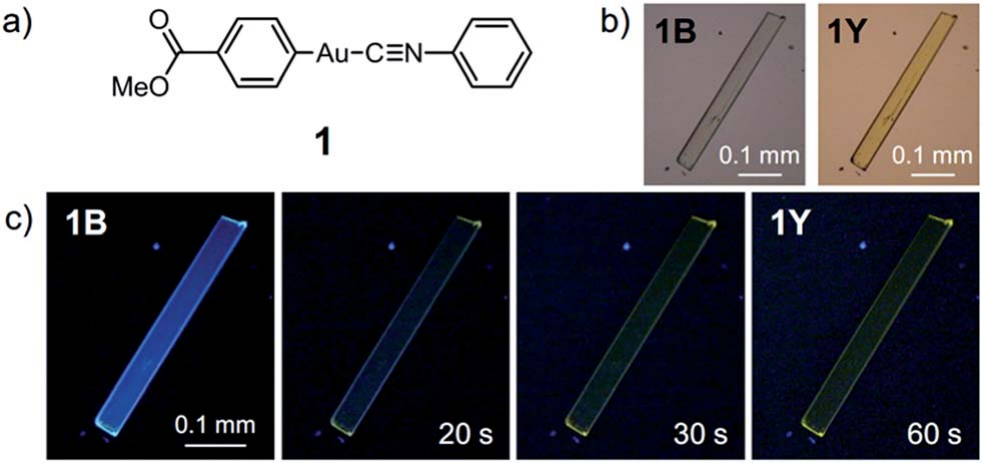
(a) The structure of complex **1**. (b) Photographs of **1B** and **1Y** under ambient light. (c) A series of photographs of the SCSC phase transition from **1B** to **1Y** induced by strong photoirradiation (367 nm, approx. 100 mW cm^–2^) taken under excitation at 365 nm.

Here, we report the first photoinduced SCSC phase transition of a gold(i) complex that is accompanied by a decrease of the Au···Au distance. To the best of our knowledge, this report is also the first observation of solid-state photochromism in a gold complex.^16^ The colorless **1B** phase, a crystallized form of the gold(i) isocyanide complex **1**, exhibits blue photoluminescence (Fig. 1). Strong UV irradiation transforms **1B** to the yellow **1Y** phase, which exhibits weak yellow emission upon excitation (Fig. 1b and c). X-ray diffraction (XRD) analyses confirmed the SCSC phase transition from **1B** to **1Y**. A comparison of these crystal structures revealed the shortening of the distance between the Au atoms after the photoinduced SCSC phase transition. It is proposed that the SCSC phase transition of **1** proceeds through tightening of the aurophilic bonds in the excited state, which has not been observed previously. Indeed, the DFT calculations based on the crystal structures indicate that the shortening of the Au···Au distance caused the red-shifted emission in the **1Y** phase generated after the photoirradiation of **1B**. In addition, **1B** exhibits a surprising “*photosalient effect*” upon irradiation with stronger UV light. To our knowledge, this report is the first observation of the photosalient effect which occurs through phase transition between polymorphs without chemical transformation or configurational alternations.^17^

## Results and discussion

### Photoinduced single-crystal-to-single-crystal phase transition

**Scheme 1:**
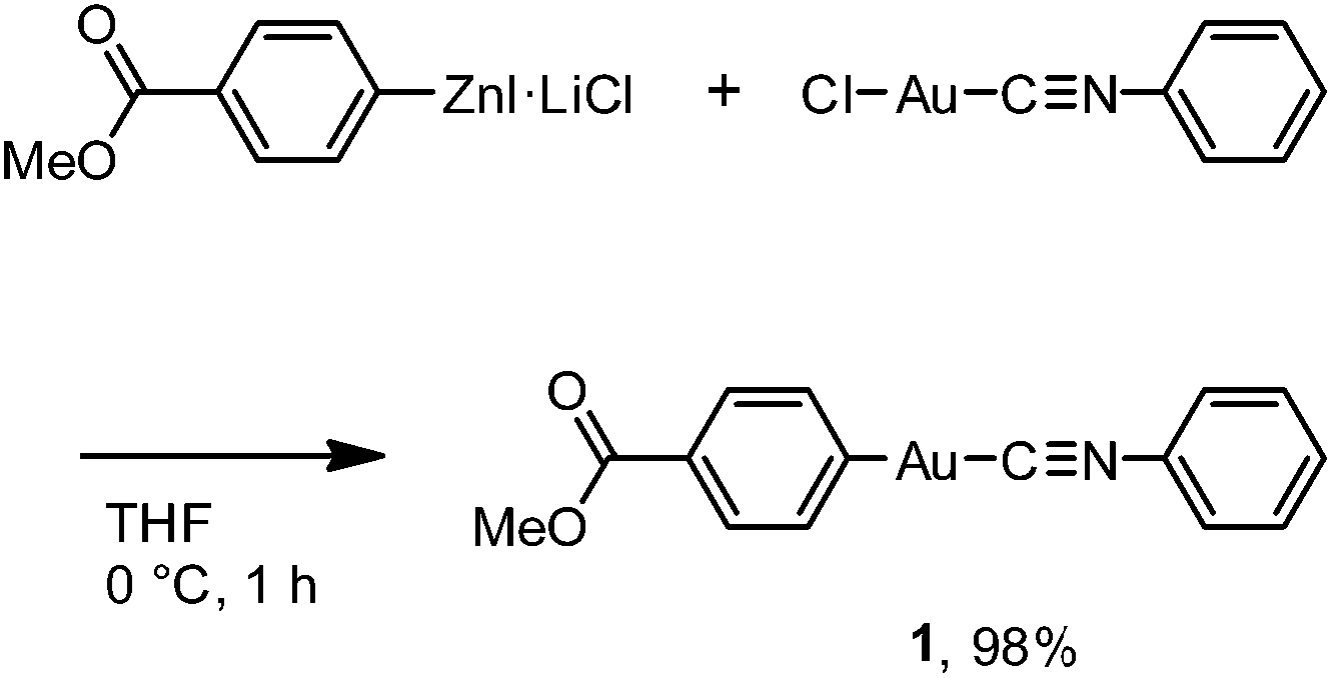
Synthesis of **1**.

Complex **1** was prepared by modification of our reported procedures (Scheme 1).^13,18^ Briefly, the (4-methoxycarbonylphenyl)zinc(ii) iodide reagent^19^ was mixed with chloro(phenyl isocyanide)gold(i) at 0 °C under a nitrogen atmosphere for 1 h to afford analytically pure **1** in 98% yield after subsequent purification by column chromatography and recrystallization from CH_2_Cl_2_/hexane. **1** was fully characterized using ^1^H and ^13^C NMR spectroscopy, high-resolution mass spectrometry and elemental analysis [see the ESI†]. The monomer of **1** in CH_2_Cl_2_ shows photoluminescence in the UV region and does not exhibit photochromism (Fig. S1 and S2†).

**Fig. 2:**
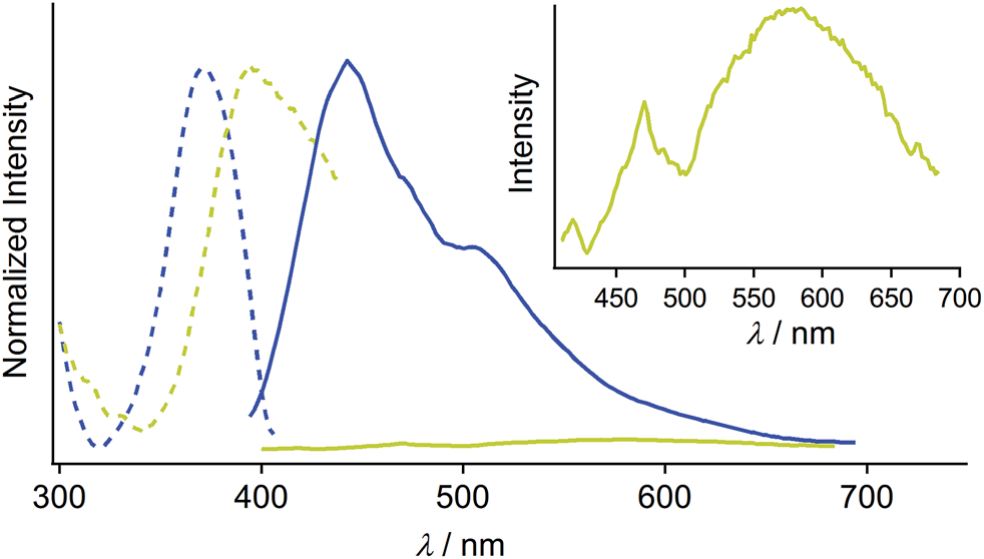
Normalized excitation (blue dashed line, detected at 450 nm) and emission (blue solid line, *λ*_ex_ = 370 nm) spectra of **1B**. Normalized excitation (greenish yellow dashed line, detected at 590 nm) and emission (greenish yellow solid line, *λ*_ex_ = 390 nm) spectra of **1Y**. The inset shows a magnified emission spectrum of **1Y**.

Crystallization of complex **1** from CH_2_Cl_2_/hexane gave the blue-emitting polymorph **1B** (Fig. 1c). The photoluminescence spectrum of **1B** obtained upon excitation at 370 nm shows an emission band ranging from 400 to 700 nm with a maximum at 448 nm (blue solid line in Fig. 2).^20^ The absolute luminescence quantum yield *Φ*_em_ and average luminescence lifetime *τ*_av_ of **1B** were determined to be 2.2% and 34.2 μs, respectively (Fig. S3 and Table S2†). The excitation spectrum of **1B** detected at 450 nm confirmed the contribution from the absorption band at 371 nm (blue dashed line in Fig. 2). Both excitation and emission bands of **1B** are red shifted from those of monomeric **1** in solution because of the crystal packing in the solid phase.

**Fig. 3:**
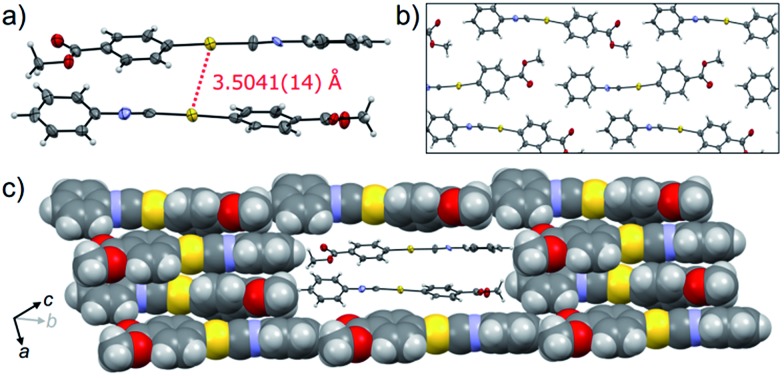
The single-crystal structure of **1B**. (a) ORTEP representation of a dimer unit. (b) ORTEP and (c) space-filling representations of the packing structure viewed along the directions roughly parallel (in b) and perpendicular (in c) to the aurophilic bonding axis. A dimer unit in (c) is shown as an ORTEP diagram to aid visualization. The crystallographic axes *a*, *b*, and *c* are depicted by arrows.

The crystalline structure of the polymorph **1B** was characterized using single-crystal X-ray analysis. A single crystal of **1B** suitable for the XRD required crystallization in the dark.^21,22^ **1B** crystallized in the triclinic space group *P*1 [*R*_1_ = 8.67%, w*R*_2_ = 24.96%, GOF = 1.106, *a* = 7.381(2) Å, *b* = 11.755(2) Å, *c* = 15.940(3) Å, *α* = 102.912(5)°, *β* = 92.025(5)°, *γ* = 100.595(5)°, *Z* = 4, *V* = 1320.8(4) Å^3^, *D*_calc_ = 2.189 g cm^–3^] (Table 1 and S3†). In many cases, the *R*_1_ and w*R*_2_ values observed in **1B** were slightly worse than those observed in **1Y**, even when the same crystal was used for **1B** and **1Y**. This result could be attributed to the small contamination of the photoinduced phase **1Y** in the original **1B**. X-ray, elemental and thermal gravimetric analyses and ^1^H NMR measurements revealed that no solvent was included in the crystalline lattice of **1B** (Fig. S5 and S6 and Table S3 and S4†). There are two crystallographically independent molecules in the lattice that exhibit dihedral angles of 26.76° and 56.04° between two intramolecular benzene rings (Fig. 3a). These molecules form dimers with a head-to-tail orientation and an intermolecular Au···Au distance of 3.5041(14) Å, indicating the formation of a very weak aurophilic bond.^7,8^ These dimers further stack with a longer Au···Au distance of 4.451(2) Å and contact with adjacent columns to form flat sheet-like layer structures (Fig. 3b and c).

**Table 1:** Summary of the single crystal data of **1B** and **1Y**

	**1B**	**1Y**
CCDC name	CCDC 987280	CCDC 987281
Crystal system	Triclinic	Triclinic
Space group	*P*1 (#2)	*P*1 (#2)
*a*/Å	7.381(2)	6.0552(5)
*b*/Å	11.755(2)	7.0297(6)
*c*/Å	15.940(3)	15.969(2)
*α*/°	102.912(5)	96.315(3)
*β*/°	92.025(5)	93.979(3)
*γ*/°	100.595(5)	90.279(3)
*V*/Å^3^	1320.8(4)	673.9(1)
*Z* Value	4	2
*V*/*Z*/Å^3^	330.2	337.0
*D*_calc_/g cm^–3^	2.189	2.145
Residual *R*_1_ ^*a*^/%	8.67	5.42
Residual w*R*_2_ ^*b*^/%	24.96	13.30
GOF^*c*^	1.106	1.045

^*a*^For data with *I* > 2.00*σ*(*I*).

^*b*^For all reflection data.

^*c*^Goodness of Fit. Residual electron density is mostly located near Au atoms because Au atoms have many electrons. See ESI Table S3 for more details.

The photophysical properties of the polymorph **1B** were altered upon photoirradiation. Upon photoirradiation of **1B** using a fluorescence microscope equipped with an ultrahigh-pressure mercury lamp (367 nm, approx. 100 mW cm^–2^) for 60 s at room temperature, the emission color of the crystals gradually changed from blue to yellow because of the formation of the polymorph **1Y** (Fig. 1c). During this process, the diffraction pattern also gradually changed (Fig. S7†), and the transparency of the crystal was retained (Fig. 1b and S8†). UV light with a lower power density necessitates a longer irradiation time to induce a transformation from **1B** to **1Y** (Table S1†). As shown in Fig. 2, the intensity of the emission from **1B** decreases under strong UV irradiation and becomes almost zero after 60 s under strong UV irradiation. The resulting **1Y** phase exhibits weak emission with a structureless maximum at 580 nm (inset in Fig. 2). This band is red shifted from that of **1B** by 132 nm. The polymorph **1Y** exhibits a very low *Φ*_em_ of ∼0.5% (Table S2†). The emission lifetime *τ*_av_ of **1Y** is 0.685 μs, which is a decrease of ∼2% upon phase transition (Fig. S3 and Table S2†). The excitation spectrum of **1Y** detected at 590 nm revealed the contribution from the absorption band at 394 nm (greenish yellow dashed line in Fig. 2). Because the excitation maximum is red shifted by 23 nm upon phase transition, the crystal becomes yellow (Fig. 1b).

**Fig. 4:**
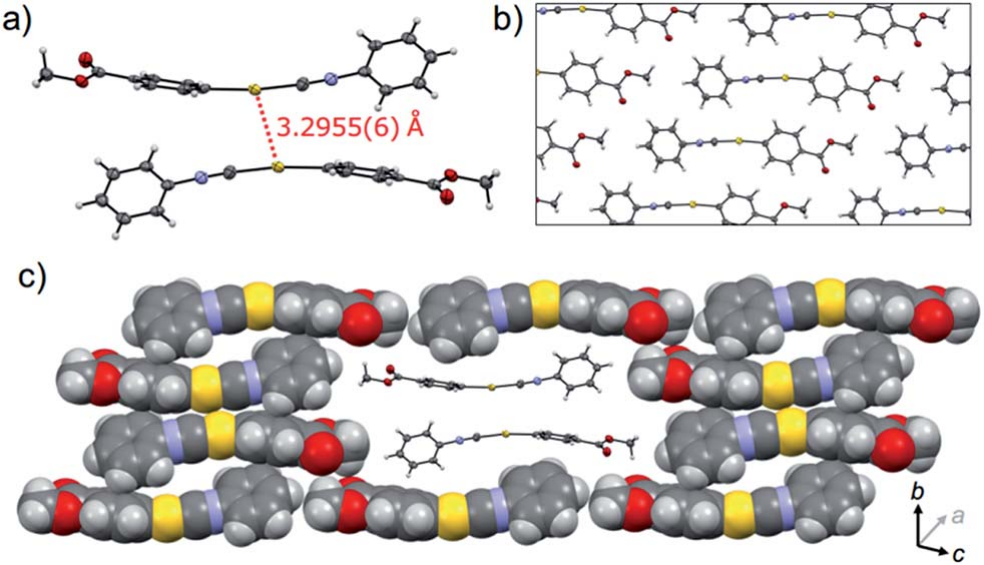
The single-crystal structure of **1Y**. (a) ORTEP representation of a dimer unit. (b) ORTEP and (c) space-filling representations of the packing structure viewed along the directions roughly parallel (in b) and perpendicular (in c) to the aurophilic bonding axis. A dimer unit in (c) is shown as an ORTEP diagram to aid visualization. The crystallographic axes *a*, *b*, and *c* are depicted by arrows.

XRD analyses indicate that **1Y** is formed through the SCSC phase transition of **1B**. The photoinduced SCSC phase transition reproducibly occurred from single crystals of **1B** to give **1Y** crystals of suitable quality for single-crystal X-ray analysis. After the single crystal X-ray analysis of **1B**, it was photoirradiated (367 nm, approx. 100 mW cm^–2^, 60 s) to yield a crystal of **1Y**, and this sample was of sufficient quality for single crystal X-ray analysis [*P*1, *R*_1_ = 5.42%, w*R*_2_ = 13.30%, GOF = 1.045, *a* = 6.0552(5) Å, *b* = 7.0297(6) Å, *c* = 15.969(2) Å, *α* = 96.315(3)°, *β* = 93.979(3)°, *γ* = 90.279(3)°, *Z* = 2, *V* = 673.9(1) Å^3^, *D*_calc_ = 2.145 g cm^–3^] (Table 1 and S3†). X-ray, elemental, and thermal gravimetric analyses and ^1^H NMR measurements revealed that decomposition of these compounds does not take place and that solvent is not included (Fig. S5 and S6 and Table S3 and S4†). **1Y** has a triclinic space group *P*1. Like **1B**, the molecules form dimers with a head-to-tail orientation. The intermolecular Au···Au distance in the dimer of **1Y** is 3.2955(6) Å, which is a marked decrease of *ca.* 0.2 Å upon crystalline structural transformation. This shorter distance indicates a stronger aurophilic bond in **1Y** compared with that in **1B**. The dihedral angle between the two benzene rings in a molecule is 47.01° (Fig. 4a). The isocyanide benzene ring participates in weak CH/π interactions with the adjacent benzene ring on the Au atom. The molecules adopt a severely distorted conformation: the structures of the –Au–C≡N– moieties deviate markedly from linearity, unlike in **1B**. The thermodynamic destabilization arising from the distortion of the molecule is probably compensated for by the formation of aurophilic bonds and weak CH/π interactions in the solid lattice (Fig. 4b and c).

### Mechanistic studies of the photoinduced single-crystal-to-single-crystal phase transition

**Fig. 5:**
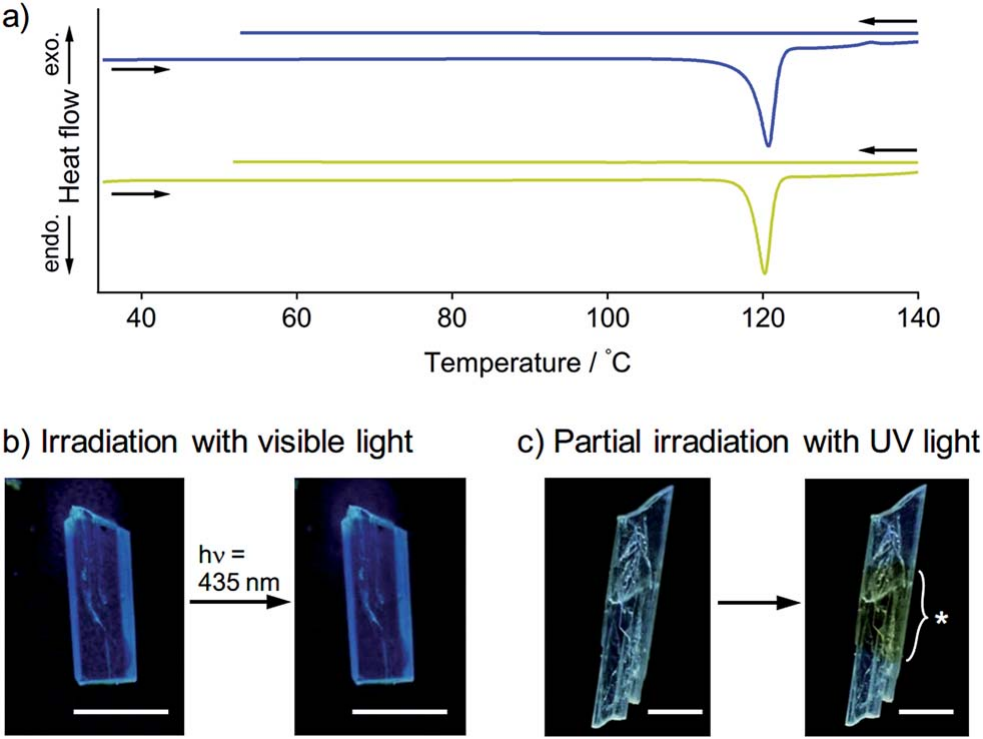
(a) DSC profiles of **1B** (blue line) and **1Y** (greenish yellow line) at heating and cooling rates of 10 and 2 °C min^–1^, respectively. (b) Photographs of the crystal **1B** before and after strong photoirradiation at 435 nm for 5 min (approx. 200 mW cm^–2^). These photographs were taken under weak UV light at 365 nm (approx. 1 mW cm^–2^). The scale bars are 0.1 mm. These photographs show that the emission color is unchanged after irradiation with visible light. (c) Photograph of a crystal **1B** obtained after photoirradiation (367 nm, approx. 100 mW cm^–2^) at a local area taken under UV light. The mark “*” denotes the irradiation region of the crystal. The scale bars are 0.1 mm.

To obtain further insights into the SCSC phase transition of **1**, a series of control experiments was performed. Upon heating, neither **1B** nor **1Y** showed thermal polymorph transformation, and both chemically decomposed at *ca.* 120 °C (Fig. S9†). The corresponding endothermic peaks were found in the differential scanning calorimetry profiles at 121 °C for **1B** and 120 °C for **1Y**, and no other peaks were observed even in the cooling profiles (Fig. 5a). This result confirms the absence of a thermal phase transition between **1B** and **1Y**. Thermal analyses using a thermography camera also confirmed that the photogenerated heat was negligible (less than 2 °C, Fig. S10 and S11†). We also confirmed that the photoinduced SCSC phase transition of **1B** was not initiated by visible light (435 nm, approx. 200 mW cm^–2^, Fig. 5b and S12†). Moreover, upon UV irradiation (367 nm, approx. 100 mW cm^–2^) of a local area of a **1B** crystal, the phase transition occurred only in the photoirradiated area and did not expand into the unirradiated region (Fig. 5c), indicating the absence of a “domino-like” mechanism.^13^ These control experiments confirmed that the phase transition of **1B** was induced by the UV photoexcitation of the crystals.

**Fig. 6:**
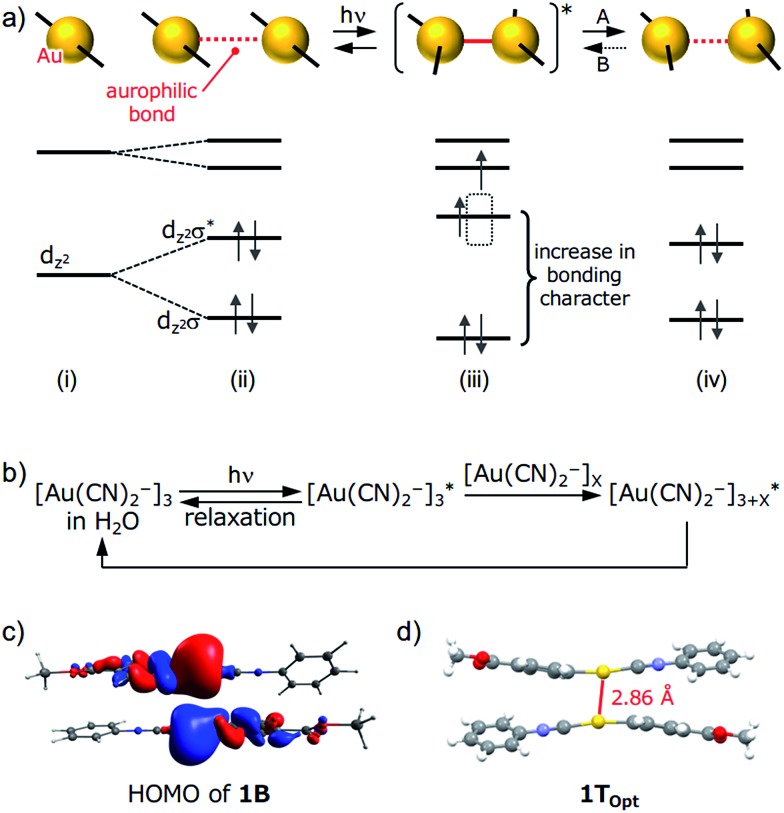
(a) A schematic representation of aurophilic bonds and their simplified orbital levels. An aurophilic bond produces d_*z* ^2^_σ and d_*z* ^2^_σ* orbitals and shortens upon photoexcitation. (b) A schematic representation of the relaxation pathway upon photoirradiation of [Au(CN)_2_^–^]_*n*_ complexes. See ref. 15 for details. (c) HOMO of the dimer derived from the single-crystal structure of **1B** (PBEPBE/SDD). (d) The structure of **1T_Opt_** obtained using triplet excited-state optimization of a **1B** dimer in a vacuum (PBEPBE/SDD).

A schematic representation of aurophilic bonds and their simplified orbital levels is shown in Fig. 6. Aurophilic interactions arising from the correlation and relativistic effects of Au atoms produce filled d_*z* ^2^_σ and d_*z* ^2^_σ* molecular orbitals and result in decreased excitation energy [(i) and (ii) in Fig. 6a].^7^ Aurophilic bonds shorten in the excited state because of the electronic transition from the filled antibonding d_*z* ^2^_σ* orbital located on the aurophilic bond to an empty orbital of higher energy [(iii) in Fig. 6a]. Although theoretical studies on this topic have been reported,^14^ it has seldom been observed experimentally. Tahara's group observed aurophilic bond shortening using time-resolved spectroscopy of aqueous solutions of [Au(CN)_2_^–^]_*n*_ complexes.^15^ In this study, the trimeric species [Au(CN)_2_^–^]_3_ predominantly present in the ground state associated into larger oligomers upon photoexcitation through the excited [Au(CN)_2_^–^]_3_* state with shortening of the aurophilic bonds (Fig. 6b). However, in this case, the original ground state recovered when photoirradiation ceased (Fig. 6b) because the molecules have enough mobility in aqueous solution to reorient to their original structure.

Although the detailed mechanism for the photoinduced SCSC phase transition of **1** is still unknown, we can provide some mechanistic insights by computational investigation (Fig. 6). A DFT calculation (PBEPBE/SDD) of the dimer of **1** derived from the single-crystal structure of **1B** possesses a HOMO with antibonding character located between the two Au atoms even though the separation between the Au atoms is on the limit of an aurophilic bond (Fig. 6c). TDDFT calculations based on the crystal structures of **1B** and **1Y** qualitatively matched the experimental excitation and emission spectra (see the ESI and Fig. S14†). Because the structural optimization of the crystal structures of **1B** and **1Y** is very difficult in terms of the computational cost, quantitative estimations of the thermodynamic stability and luminescence properties could not be achieved. Geometry optimization of the dimer derived from **1B** in the triplet state (**1T_Opt_**) in a vacuum gave a marked decrease in the Au···Au separation (2.86 Å, Fig. 6d). The structures and molecular orbitals of **1T_Opt_** are more similar to those of the dimer of **1Y** compared with those in **1B** (Fig. S13 and S15 and Table S7†). These results indicate that the structure transformation from **1B** to **1Y** can be facilitated through the photoexcited state of **1B** [(ii)–(iv) in Fig. 6a]. Although the structural conversion from the photoexcited state to **1Y** (“A” in Fig. 6a) would compete with the relaxation to the original ground state **1B**, once the phase transformation to **1Y** occurred in a local area, the reverse phase transition from **1Y** to **1B** (“B” in Fig. 6a) does not proceed thermally, probably because the movement of the molecules in the **1Y** polymorph is restricted by the relatively strong intermolecular interactions, such as CH/π interactions and aurophilic interactions (Fig. 4). Irradiation to **1Y** could not cause it to revert to **1B** because the structure of the excited state would be more similar to **1Y** than the structure of **1B**. Thus, the entire **1B** crystal eventually transformed into the new phase **1Y** after a certain period of photoirradiation. Although many studies on the transient shortening of metallophilic bonds (Pt, Rh, and Ir) in the photoexcited state have been reported, these structural changes reverted back to the original structures after photoirradiation ceased.^23^ The present study is the first example of a photoinduced SCSC transformation with a shortening of metallophilic bonds.

### The first photosalient effect induced by a phase transition between polymorphs

Recently, a rare dynamic phenomenon of organic crystals, the “salient effect”, has been attracting much attention.^17^ The thermosalient effect, in which organic crystals jump after the crystal is heated, was first reported in 1983.^24^ A related phenomenon in which a crystal jumps due to irradiation with light is referred to as the “photosalient effect”.^17^ The “salient effect” is produced by the internal crystal constraints imposed by changes in the crystalline shape and/or volume at the interface between the two phases upon the thermo- or photoinduced crystalline structural change. Recently, intense explorations of thermo- and photosalient effects have been made by Naumov and colleagues;^17,25,26^ however, compounds that show the photosalient effect are still very rare.^17,26^

**Fig. 7:**
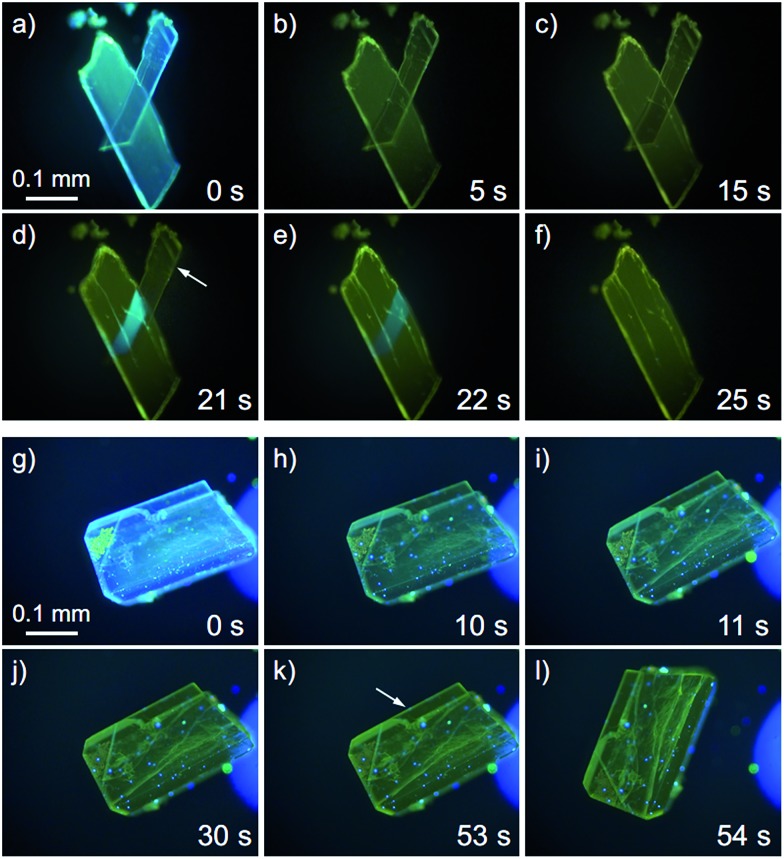
A series of photographs of the photosalient effect of **1B** through the transformation into **1Y** induced by strong photoirradiation (367 nm, approx. 400 mW cm^–2^). Photographs from (a) to (f) and (g) to (l) were cropped from the ESI Movies S1 and S2,† respectively. The arrows in (d) and (k) indicate the crystals just before the jump. The irradiation times are shown.

We found that the **1B** crystals show the “salient effect” upon strong UV light irradiation (367 nm, approx. 400 mW cm^–2^). As shown in Fig. 7a–f and the ESI Movie S1,† the blue emission of the surface of the **1B** crystal becomes yellow after 5 s of photoirradiation through the phase transition from **1B** to **1Y** (see Fig. 7a and b), and after 21 s the crystal jumps (see Fig. 7c–e). Not all crystals of **1B** showed this jumping phenomenon; approximately 1% of the irradiated crystals of **1B** jumped (see also the ESI Movie S2 and Fig. 7g–l and S16†) and approximately 10% of the irradiated crystals split (ESI Movie S3 and Fig. S17†). Furthermore, more than 80% of the irradiated crystals showed cracking (Fig. S18†). These mechanical responses upon photoirradiation suggested that the transformation from **1B** to **1Y** is a martensitic-type transition, similar to most salient-active materials, rather than a phase transition through a nucleation–elongation mechanism. In Naumov's articles,^17,25^ the authors discussed the idea that the thermosalient phase transition generally occurs between two phases with an identical symmetry and space group. Thus, the overall packing and unit cell parameters of the two phases are only slightly, yet distinctly changed upon applying the external stimuli. This notion can also be applied to the photosalient phase transition of **1** because the crystalline structures of **1B** and **1Y** fulfil these requirements: the space groups of **1B** and **1Y** are both *P*1, and their *V*/*Z* values are almost the same (**1B**: 330.2 Å^3^; **1Y**: 337.0 Å^3^, Table 1). The photosalient effect is generally induced by a photochemical reaction (chemical structural changes, *e.g.*, a [2 + 2] cycloaddition, a ring closing reaction, *etc.*).^26^ Contrary, the present study is the first example of the photosalient effect induced by a phototriggered phase transition between polymorphs (a packing structural change or molecular arrangement change) without chemical structural changes. As shown in the aforementioned control experiments, the heating effect that would accompany photoexcitation^17^ has negligible influence on the phase transition of **1** (Fig. S10 and S11†), and thus is not related to its photosalient effect. This conclusion is also supported by the fact that **1B** does not show any thermal phase change below 120 °C (Fig. 5a). The aurophilic bonding formation in the crystal was induced by light irradiation and generated the macro-scale mechanical power that can sputter the crystals.

## Conclusion

In summary, we have described the first photoinduced SCSC phase transition of the gold(i) isocyanide complex **1** with a shortening of the aurophilic bond. This phase transformation was accompanied by a defined color change under UV light. We propose that the shortening of the aurophilic bond in the excited state is the driving force behind this transition; structural relaxation from the excited state to **1Y** is followed by immobilization of the structural change, including the short aurophilic bond, which inhibits reversal to the original **1B**. The first photosalient effect induced by a polymorph transformation was also discovered. These findings could be an important lead in the design of novel photosensitive materials that exhibit reorganization of intermolecular interactions induced by photoirradiation.
