# Supplementary material for: Photoinduced single-crystal-to-single-crystal phase transition and photosalient effect of a gold(i) isocyanide complex with shortening of intermolecular aurophilic bonds
Source: Chem Sci. 2014 Dec 15;6(2):1491–7. doi: 10.1039/c4sc02676d (PMC5811136; doi:10.1039/c4sc02676d)
Supplement: Supplementary file 1 [file SC-006-C4SC02676D-s001.pdf]

Electronic Supplementary Material (ESI) for Chemical Science.
This journal is © The Royal Society of Chemistry 2014

Electronic Supplementary Information

Photoinduced Single-Crystal-to-Single-Crystal Phase Transition and Photosalient Effect of a Gold(I) Isocyanide Complex with Shortening Intermolecular Aurophilic Bonds

Tomohiro Seki, Kenta Sakurada, Mai Muromoto, and Hajime Ito*

Division of Chemical Process Engineering and Frontier Chemistry Center (FCC), Graduate School of Engineering, Hokkaido Uiversity, Sapporo, Hokkaido 060-8628, Japan

Email: hajito@eng.hokudai.ac.jp

Contents

1. General S1

2. Synthesis S2

3. Optical Properties in Solution Phase S3

4. Irradiation Time Required for the Emission Color Change under UV Light S5

5. Photophysical Properties S6

6. Data for Single Crystal X-ray Structural Analyses and Low-Temperature Luminescence S7

7. TGA, NMR Spectroscopy and Elemental Analyses of 1B and 1Y S10

8. Change in Diffraction Patterns of 1B upon Photoirradiation with Retaining Transparency S12

9. A Series of Control Experiments for Characterization of Phase Transition of 1 S13

10. DFT Calculations S16

11. Mechanical Response of 1B upon Strong Photoirradiation S23

12. References S24

13. NMR Charts S25

1. General

All commercially available reagents and solvents are of reagent grade and were used without further purification unless otherwise noted. Solvents for the synthesis were purchased from commercial suppliers, degassed by three freeze-pump-thaw cycles and further dried over molecular sieves (4 Å). NMR spectra were recorded on a JEOL JNM-ECX400P or JNM-ECS400 spectrometer (^{1}H: 400 MHz; ^{13}C: 99.5 MHz) using tetramethylsilane and $CDCl_3$ as internal standards, respectively. Emission spectra were recorded on a Hitachi F-7000 spectrometer. Fluorescence microscopic spectra were recorded on a Photonic Hamamatsu PMA-12 Multichannel Analyzer. The emission quantum yields of the solid samples were recorded on a Hamamatsu Quantaurus-QY spectrometer with an integrating sphere. Emission lifetime measurements were recorded on a Hamamatsu Quantaurus-Tau spectrometer. Elemental analyses and low- and high resolution mass spectra were recorded at the Center for Instrumental Analysis, Hokkaido University. Photographs were obtained using Olympus BX51 or SZX7 microscopes with Olympus DP72, Nikon D5100 or RICOH CX1 digital cameras. As strong UV and visible light for photoinduced phase transition of **1**, an Olympus BX51 fluorescence microscope equipped with an Ushio 100 W ultrahigh-pressure mercury lamp USH-1030L and Olympus fluorescence mirror unit U-MWU2 (λ_{max} = 367 nm, approx. 100 or 400 mW·cm^{-2}) or U-MWUV2 (λ_{max} = 435 nm, approx. 200 mW·cm^{-2}) was used. Power density of the UV light was measured on Hamamatsu UV power meter C6080-04 and C6080-385.

2. Synthesis

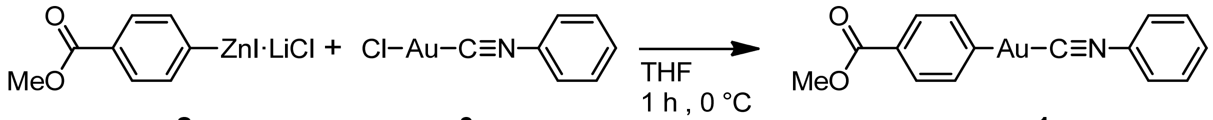

2 3 1

To chloro(phenyl isocyanide)gold(I) (**3**, 0.159 g, 0.5 mmol), THF (0.5 ml) was added under nitrogen atmosphere. After cooling to 0 °C, organozinc iodide reagent **2**[1] in THF (1.0 ml, 0.75 mmol, 0.727 M) was added dropwise with stirring. After 1 h stirring, the reaction was quenched by the addition of a phosphate buffer solution and then extracted with CH_2Cl_2 three times and washed with H_2O and brine. The organic layers were collected and dried over Na_2SO_4. After filtration, the solvent was removed *in vacuo*. Further purification by flash column chromatography (SiO_2, CH_2Cl_2/hexane = 3:1) gave a white solid. The resulting solid was dissolved in CH_2Cl_2 in a vial and hexane was carefully layered for crystallization and allowed to stand at room temperature to give analytically pure crystals of **1** (0.2126 g, 0.488 mmol, 98 %). ^{1}H NMR (400 MHz, $CDCl_3$, δ): 3.88 (s, 3H), 7.50–7.53 (m, 5H), 7.55–7.59 (m, 2H), 7.90 (d, *J* = 8.4 Hz, 2H). ^{13}C NMR (100 MHz, $CDCl_3$, δ): 51.6 (CH_3), 124.6 (*C*), 126.5 (*CH*), 126.9 (*C*), 127.6 (*C*H), 129.7 (*C*H), 130.1 (*CH*), 140.2 (*C*H), 160.5 (*C*), 167.8(*C*), 170.6(*C*). MS-FAB (*m*/*z*): $[M+H]^+$ calcd for $C_{15}H_{13}AuNO_2$, 436.0612; found, 436.0610. Anal. Calcd for $C_{15}H_{12}AuNO_2$: C, 41.39; H, 2.78; N, 3.22. Found: C, 41.34; H, 2.81; N, 3.21.

*Preparation of **1B***: The polymorph **1B** is readily obtained by crystallization. Typically, **1** (30 mg) is dissolved in 2 mL of CH_2Cl_2 in a vial and hexane (8 mL) was carefully layered. After standing at −25 ºC for a few days, colorless, blue-emitting crystals **1B** are formed. For the preparation of **1B** with an enough quality for single crystal X-ray diffraction analysis, aforementioned crystallization should be conducted under dark conditions.

*Preparation of **1Y***: The polymorph **1Y** is obtained by photoirradiation of **1B**. Typically, an Olympus BX51 fluorescence microscope equipped with an Ushio 100 W ultrahigh-pressure mercury lamp USH-1030L and an Olympus fluorescence mirror unit U-MWU2 (λ_{max} = 367 nm) without any neutral density filters is used as a strong UV light (approx. 100 mW·cm^{-2}) for photoexcitation. Photoirradiation of **1B** for 60 s under ambient condition, phase transition into **1Y** phase occurs with emission color change. Longer irradiation time sometimes results in the decomposition of **1**.

3. Optical Properties in Solution Phase

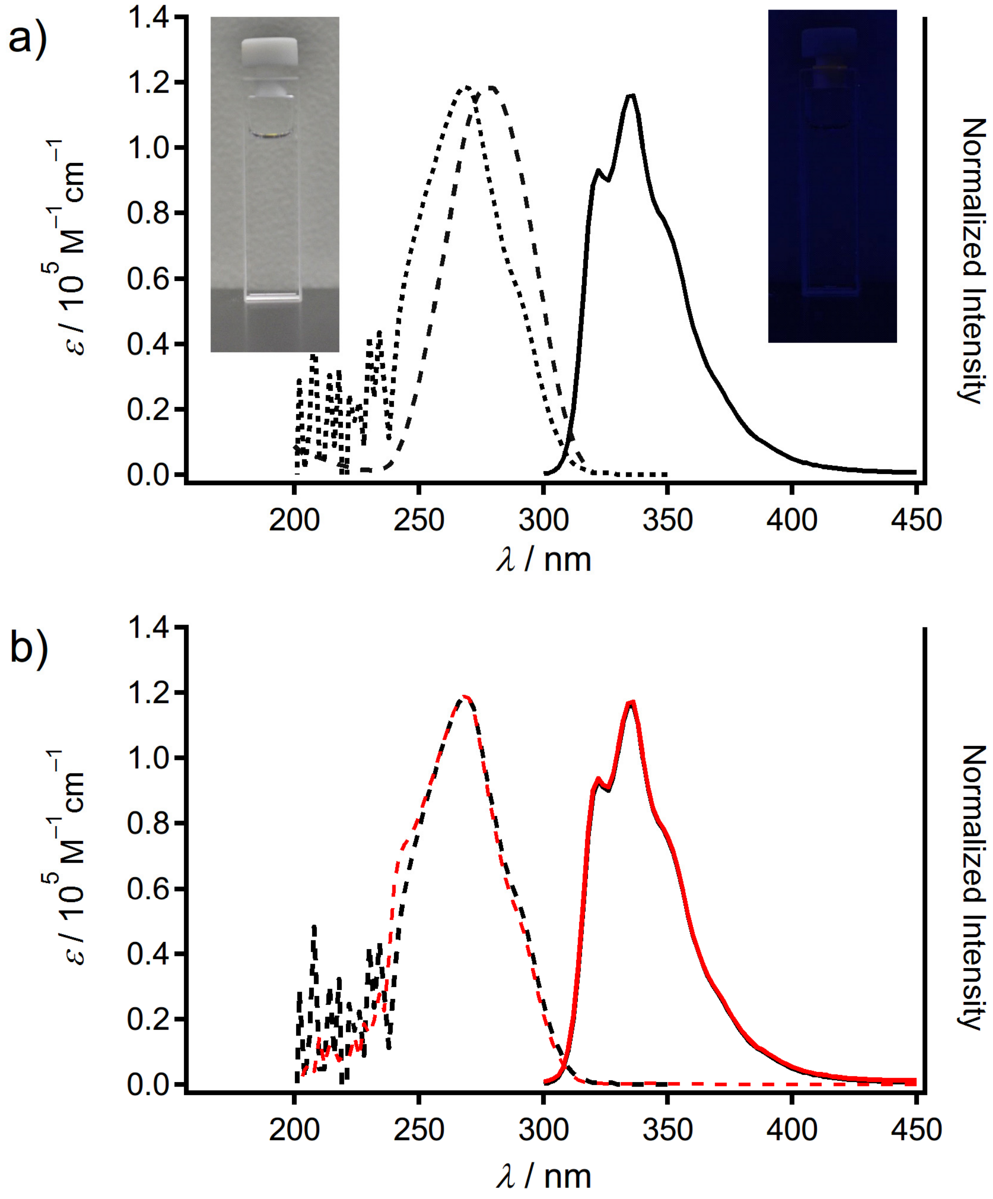

Fig. S1 a) UV/vis absorption (dotted line), excitation (dashed line, λ_{em} = 335 nm), and emission spectra (solid line, λ_{ex} = 280 nm) of **1** in CH_2Cl_2 (c = 1.7 × 10^{-6} M) at room temperature. Insets show photographs of **1** in CH_2Cl_2 taken under ambient (left) and UV light (right) were shown in inset. b) Absorption (dashed line) and emission spectra (solid line, λ_{ex} = 280 nm) of **1** in CH_2Cl_2 (c = 1.7 × 10^{-6} M) at room temperature before (black lines) and after (red lines) photoirradiation for 300 s (approx. 1 mW·cm^{-2}).

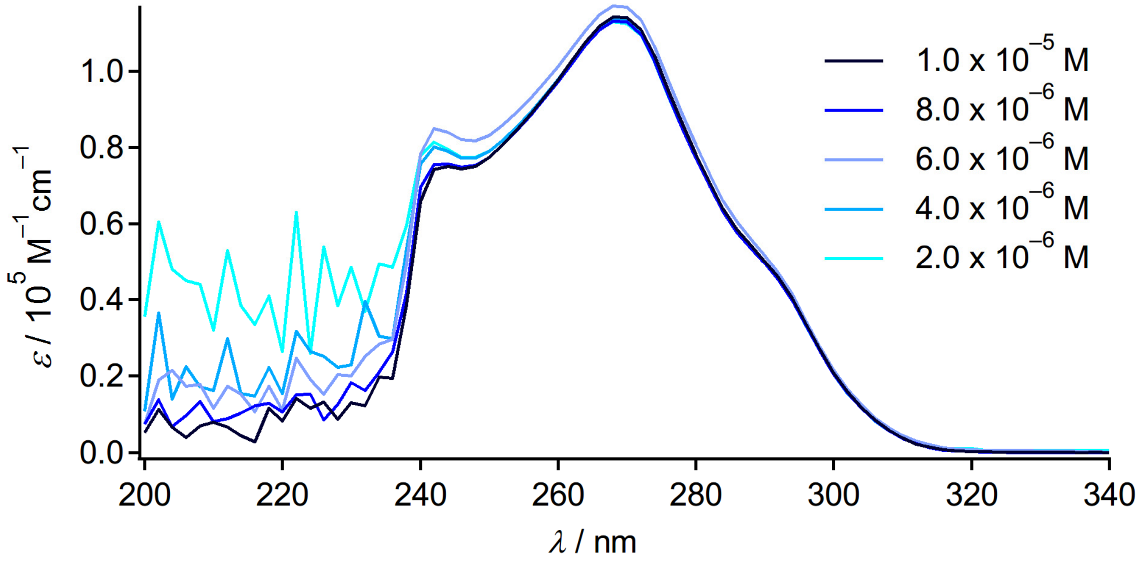

Fig. S2 Concentration-dependent UV/vis absorption spectra of **1** in CH_2Cl_2 at room temperature.

4. Irradiation Time Required for the Emission Color Change under UV Light

Table S1 Qualitative relationship between power density of the UV light (367 nm) and irradiation time required for SCSC phase transition of **1B**.

Power density /mW·cm^{-2}	Time /min
< 0.1	–[a]
5	10
25	3
100	1
400	0.1

[a]Phase transition does not take place.

Note: Irradiation time required for the phase transition from **1B** to **1Y** depends strongly on the quality of the crystals even with the same power density of UV light. However, repeated measurements qualitatively revealed that UV light with lower power density requires longer irradiation time for SCSC phase transition as shown in Table S1.

5. Photophysical Properties

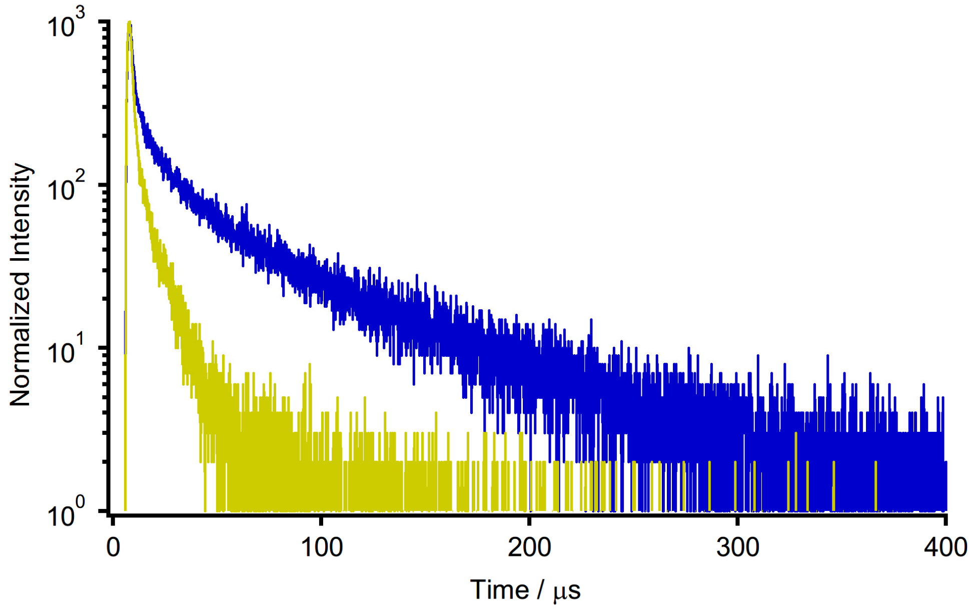

Fig. S3 Emission decay profiles of **1B** at 450 nm (blue line) and **1Y** at 580 nm (greenish yellow line) under excitation at 370 nm.

Table S2 Photophysical properties of **1**

	Φ_{em} / %	τ_{av} / μs[d,e] (λ_{em} / nm)	τ_1 / μs[d] (A / -)	τ_2 / μs[d] (A / -)	τ_3 / μs[d] (A / -)
**1B**	2.2[a]	34.2 (485)	0.292 (0.27)	5.839 (0.17)	59.079 (0.56)
**1Y**	0.5[b]	0.685 (580)	0.297 (0.79)	2.141 (0.21)	–
**Sol**[g]	22[c]	–[f] (335)	–[f]	–[f]	–[f]

[a]λ_{ex} = 370 nm. [b]λ_{ex} = 390 nm. [c]λ_{ex} = 280 nm. [d]λ_{ex} = 370 nm for **1B** and **1Y**, λ_{ex} = 280 nm for a CH_2Cl_2 solution of **1**. [e]$\tau_{av} = (A_1\tau_1 + A_2\tau_2 + \cdots) / (A_1 + A_2 + \cdots)$. [f]Curve fitting failed. [g]Data obtained from a CH_2Cl_2 solution of **1** (1.7×10^{-6} M) at room temperature.

6. Data for Single Crystal X-ray Structural Analyses and Low-Temperature Luminescence

Single crystal X-ray structural analyses were carried out on a Rigaku R-AXIS RAPID diffractometer using graphite monochromated Mo-K_{α} radiation. The structure was solved by direct methods and expanded using Fourier techniques. Non-hydrogen atoms were refined anisotropically. Hydrogen atoms were refined using the riding model. All calculations were performed using the CrystalStructure crystallographic software package except for refinement, which was performed using SHELXL-97.[2]

Confirmation that Solvent was Not Included: We checked the maximum residual electron density in **1B** and **1Y**. The maximum and minimum peaks in the final differential maps were 2.44 e^- and –3.78 e^- [$Å^{-3}$], respectively, for **1B**; 5.16 e^- and –2.80 e^- [$Å^{-3}$], respectively, for **1Y**. These values are within the range of ±0.075 e^- × Z_{max} = ±5.9 [$Å^{-3}$] for the complex **1**, where Z_{max} denotes the maximum atomic number in the lattice. This indicated that no residual electron density that could be assigned to other molecules, such as solvent, was present in the crystal structure. This is a standard analysis to identify small molecule inclusion in crystal structures.

Table S3 Summary of X-ray crystallographic data for **1B** and **1Y**.

Polymorph	**1B**	**1Y**
CCDC Name	CCDC 987280	CCDC 987281
Empirical Formula	$C_{15}H_{12}AuNO_2$	$C_{15}H_{12}AuNO_2$
Formula Weight	435.23	435.23
Crystal System	triclinic	triclinic
Crystal Size / mm	0.171 × 0.115 × 0.037	0.171 × 0.115 × 0.037
a / Å	7.381(2)	6.0552(5)
b / Å	11.755(2)	7.0297(6)
c / Å	15.940(3)	15.969(2)
α / °	102.912(5)	96.315(3)
β / °	92.025(5)	93.979(3)
γ / °	100.595(5)	90.279(3)
V / Å^3	1320.8(4)	673.9(1)
Space Group	P-1 (#2)	P-1 (#2)
Z value	4	2
Dcalc / g·cm^{-3}	2.189	2.145
Temperature / K	123	123
$2\theta_{max}$ / °	51.2	54.9
μ (MoK$_\alpha$) / cm^{-1}	111.729	109.488
No. of Reflections Measured	Total : 10450<br>Unique : 4756<br>(R_{int} = 0.1301)	Total : 5525<br>Unique : 2516<br>(R_{int} = 0.0574)
Residuals: R_1 (I > 2.00σ (I)) / %	8.67	5.42
Residuals: wR_2 (All reflections) / %	24.96	13.30
Goodness of Fit (GOF)	1.106	1.045
Maximum peak in Final Diff. Map / Å^3	2.44 e$^-$	5.16 e$^-$
Minimum peak in Final Diff. Map / Å^3	–3.78 e$^-$	–2.80 e$^-$

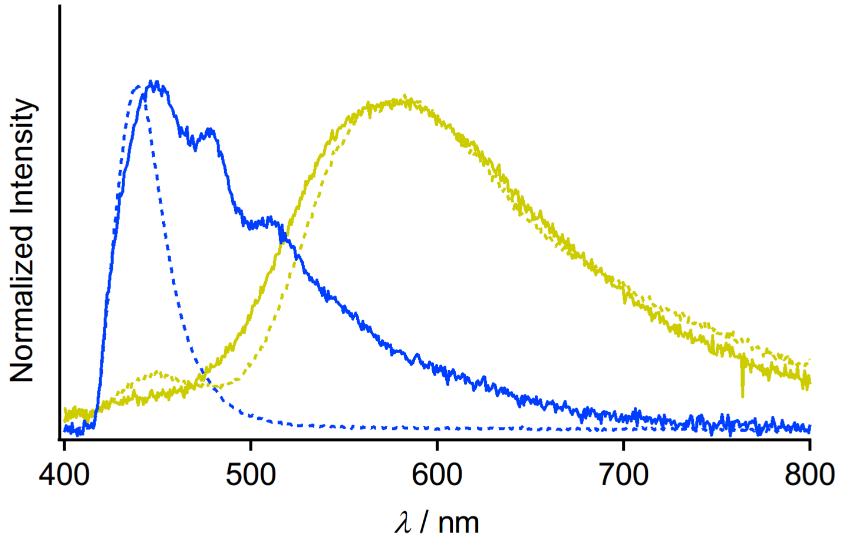

Fig. S4 Normalized emission spectra of **1B** (blue lines) and **1Y** (greenish yellow lines) at room temperature (solid lines) and 123 K (dotted lines) with an UV light at 367 nm with a power density of approx. 3 mW·cm^{-2}.

Note: Emission maxima of **1B** and **1Y** are almost unchanged upon decreasing temperature to 123 K. This indicates that lowering temperature of **1B** and **1Y** does not induce their polymorph transformation.

7. TGA, NMR Spectroscopy and Elemental Analyses of 1B and 1Y

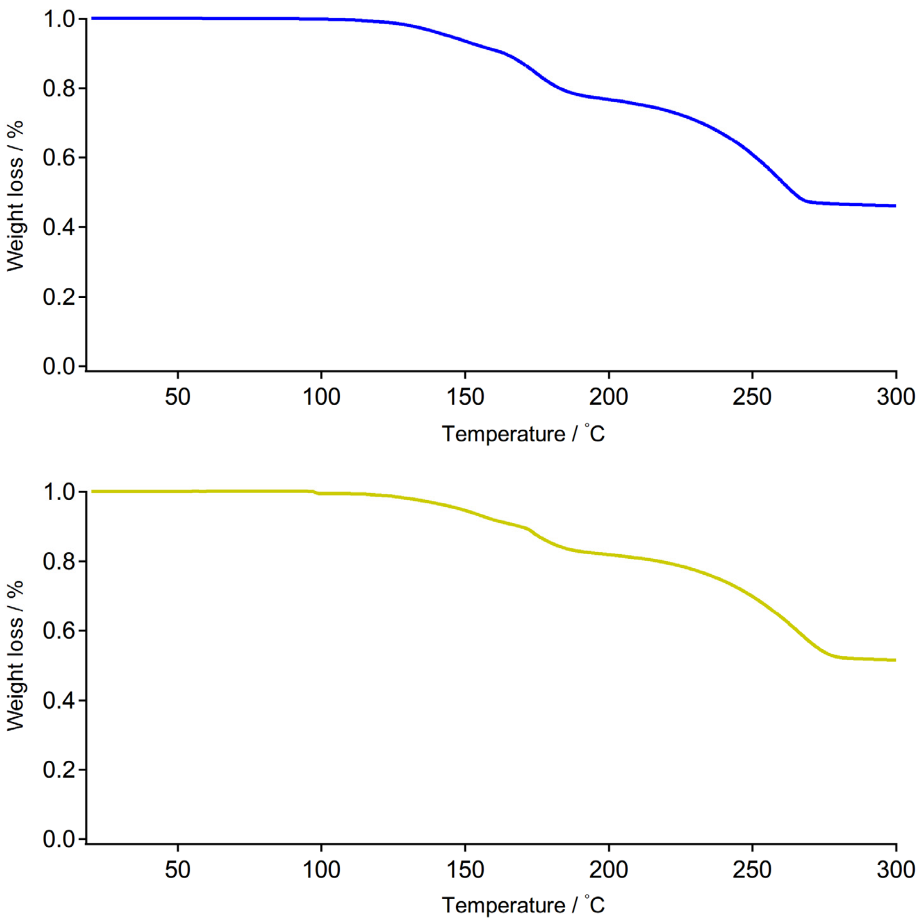

Fig. S5 TGA profiles of **1B** (blue line) and **1Y** (greenish yellow line) at a heating rate of 10 °C min^{-1}.

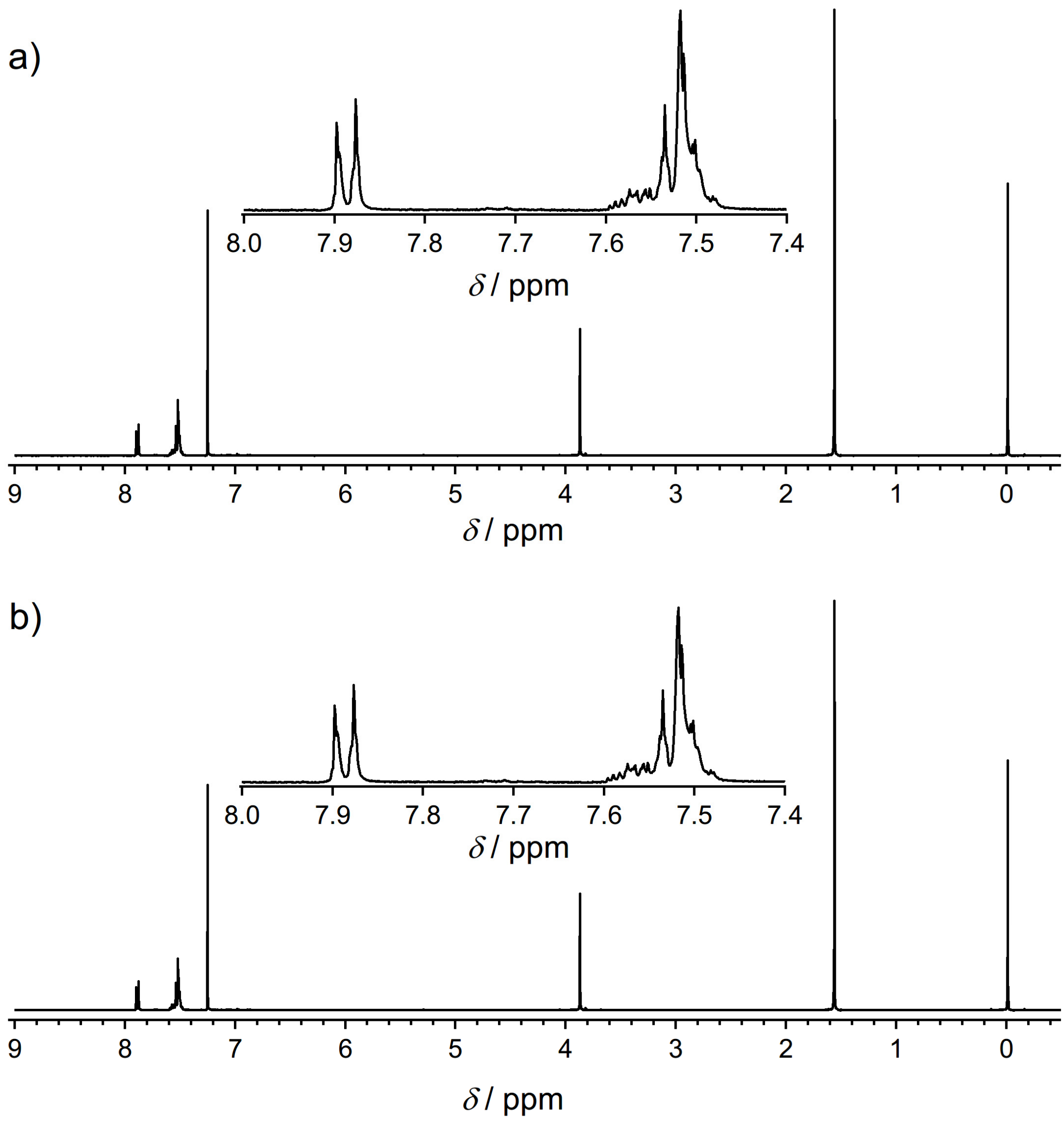

Fig. S6 ^1H NMR spectra of **1B** (a) and **1Y** (b) dissolved in $CDCl_3$.

Table S4 Elemental analyses of the polymorphs **1B** and **1Y**.

	C	H	N
Calculated for **1** ($C_{15}H_{12}AuNO_2$)	41.39	2.78	3.22
**1B**	41.34	2.81	3.21
**1Y**	41.32	2.82	3.23

8. Change in Diffraction Patterns of 1B upon Photoirradiation with Retaining Transparency

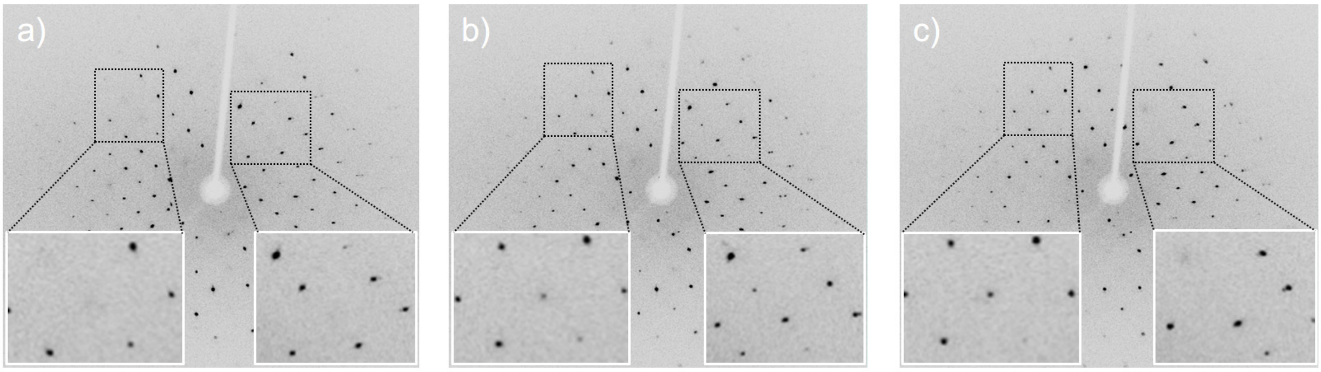

Fig. S7 Photoinduced changes of the diffraction patterns of **1B**: photoirradiation (367 nm, 100 mW·cm^{-2}) times are 0 s for a), 30 s for b), and 3 min for c). Insets show magnified patterns exhibiting gradual change of the diffractions upon photoinduced phase transition from **1B** to **1Y**.

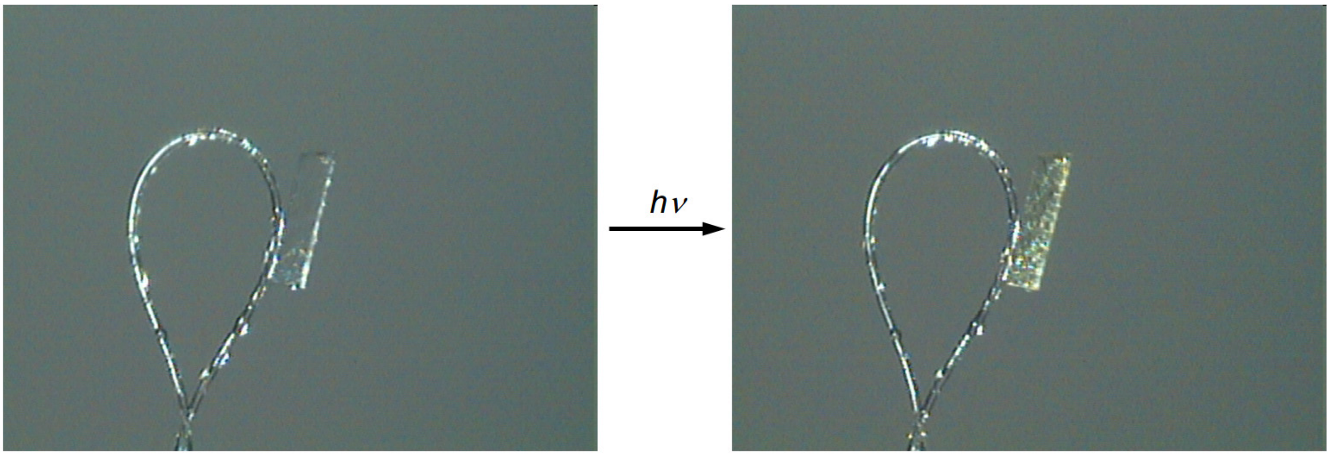

Fig. S8 Photographs of **1B** (left) and **1Y** (right) under ambient light. These samples were obtained before and after irradiation of strong UV light (367 nm, approx. 100 mW·cm^{-2}), respectively.

9. A Series of Control Experiments for Characterization of Phase Transition of 1

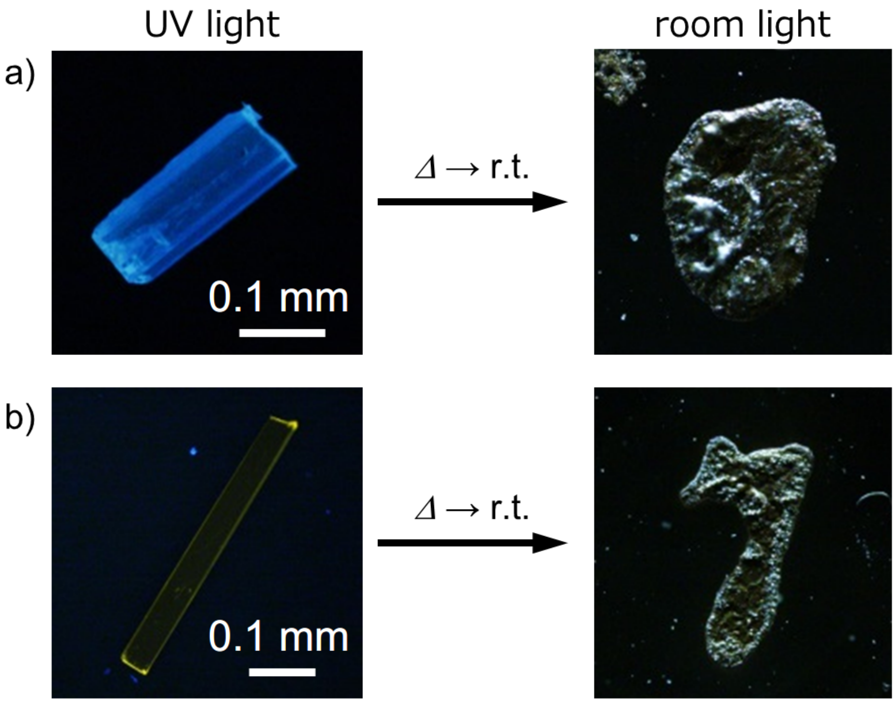

Fig. S9 Left: Photographs of **1B** a) and **1Y** b) under UV irradiation. Right: Photographs of the same crystals after decomposition at 120 °C and then cooling to room temperature taken under ambient light.

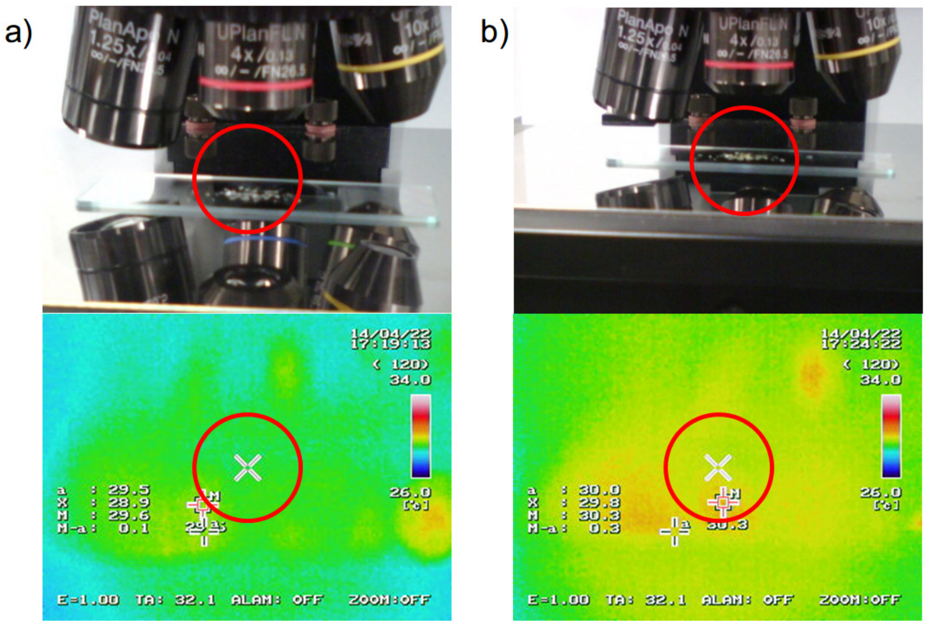

Fig. S10 Photographs (upper) and thermography (lower) of crystals of **1** on glass plate before a) and after b) photoinduced phase transition from **1B** to **1Y** by UV irradiation (***5 min, 367 nm, approx. 100 mW·cm***$^{-2}$). Red circles indicate position of crystals. Temperature raise upon photoirradiation is 0.9 °C, indicating negligible influence of heat on photoinduced phase transition.

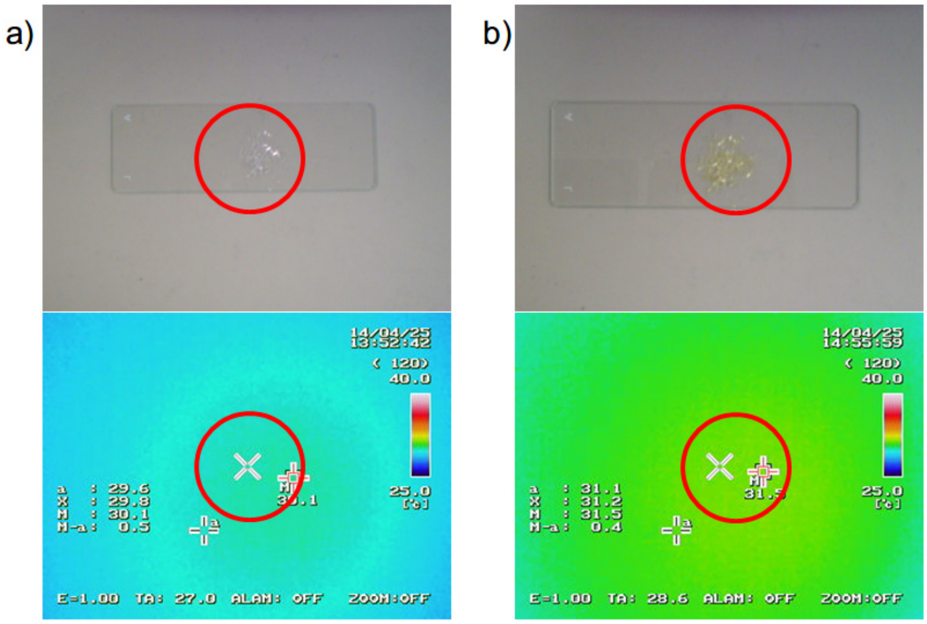

Fig. S11 Photographs (upper) and thermography (lower) of crystals of **1** on glass plate before a) and after b) photoinduced phase transition from **1B** to **1Y** by UV irradiation (***1 h, 365 nm, approx. 3 mW·cm***$^{-2}$). Red circles indicate position of crystals. Temperature raise upon photoirradiation is 1.4 °C, indicating negligible influence of heat on photoinduced phase transition.

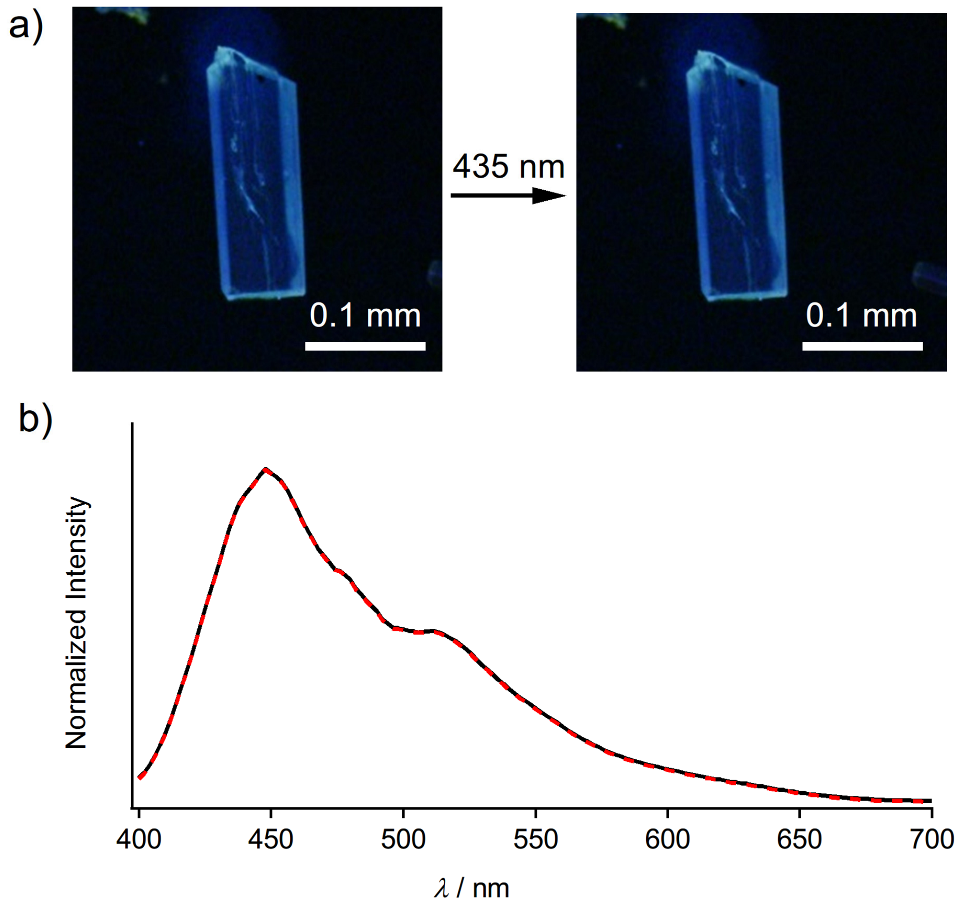

Fig. S12 a) Photographs of the crystal **1B** before and after photoirradiation at 435 nm for 5 min (approx. 200 mW·cm^{-2}) taken under UV light at 365 nm. b) Emission spectra (λ_{ex} = 365 nm) of **1B** before (black solid line) and after photoirradiation (red dashed line) at 435 nm (approx. 200 mW·cm^{-2}).

10. DFT Calculations

All calculations were performed using the Gaussian 09W (revision C.01) and Gaussian 09 program package.[3] In the calculations, the SDD basis set with an effective core potential was used for Au and other atoms. PBEPBE functionals were used because other functionals such as B3LYP, CAM-BLYP, LC-BLYP and M06 did not well reproduce the experimental results.[4–7] The geometry of the dimeric structures of **1B** and **1Y** were calculated using coordinates of C, N, and Au taken from the corresponding X-ray structures. The positions of heavy atoms were fixed and only the positions of H atoms were optimized using the Spartan '10 MMFF force-field calculation.[8] Molecular orbitals were drawn using the Avogadro 1.1.0 program.[9]

The triplet-state structure optimizations of the dimers taken from the crystal structures of **1B** and **1Y** were carried out in vacuum, resulting in the same structure, $\mathbf{1T_{opt}}$ (Figure S13). The structure of $\mathbf{1T_{opt}}$ is more similar to that of **1Y** than that of **1B**. Because the optimization was carried out in vacuum, these results indicate that the photoexcited molecules in the **1B** crystals are forced to transform to the excited state-related structure **1Y**. This also suggests that the triplet $\mathbf{1T_{opt}}$ structure is close to the excited state structure of **1Y**. A change in molecular structure induced by photoexcitation does not generally occur readily in crystals. Thus, the structure of the photoexcited molecules in a **1B** crystal is similar to the ground-state structure of **1B**. In contrast, the structure of the photoexcited molecules in **1Y** crystals is similar to the ground-state structures of **1Y** and $\mathbf{1T_{opt}}$.

The excitation spectra for **1B** (λ_{max} = 371 nm) and **1Y** (λ_{max} = 394 nm) crystals were reproduced qualitatively by TDDFT calculations using the dimer structures derived from the X-ray structures of **1B** and **1Y** (Figure S15, Table S5 and S6). The lowest singlet excited state (1B-S_1, Table S5) for **1B** includes the antibonding (HOMO) and bonding (HOMO−2 and HOMO−3) orbitals along two Au atoms, and π* orbitals of isocyanide (LUMO and LUMO+1). The higher singlet excited states (1B-S_2, 1B-S_3) also show a mixed character including bonding (HOMO−2, LUMO, and LUMO+2), antibonding (HOMO−5, HOMO−4, HOMO−3, HOMO−1 and HOMO), π* of isocyanide (LUMO+1), and delocalized (LUMO+4 and LUMO+7) orbitals. The lowest singlet excited state (1Y-S_1, Table S6) of **1Y** includes the bonding (HOMO−2, LUMO, and LUMO+2) and antibonding (HOMO, HOMO−3, and HOMO−4) orbitals along two Au atoms, and π* orbitals of isocyanide (LUMO+1). The higher singlet excited states (1Y-S_2, 1Y-S_3) also show a mixed character including bonding (HOMO−2 and LUMO), antibonding (HOMO−6, HOMO−5, HOMO−4, HOMO−3, and HOMO−1), and π* orbitals of isocyanide (LUMO+1).

The trend in the phosphorescence spectra of **1B** and **1Y** could be qualitatively reproduced by DFT calculations. The phosphorescence spectrum of **1B** (λ_{max} = 448 nm) matches the vertical excitation energy (Table S7, 1B-T_1, λ = 491 nm) calculated by TDDFT of the dimer structure taken from the **1B** crystal-structure analysis. The lowest triplet excited state includes antibonding HOMO and bonding LUMO, indicating that the aurophilic bond tends to be strong in the triplet excited state compared with in the ground state. Although the phosphorescence of **1Y** (λ_{max} = 580 nm) is different from the calculated vertical excitation energy (Table S7, 1Y-T_1, λ = 463 nm), the red shift from the

phosphorescence of **1B** qualitatively corresponds to the T_1 energy (Table S7, 1ST-T_1, λ = 664 nm) of the TDDFT calculation of the singlet model structure (**1ST$_{opt}$**) with **1T$_{opt}$** geometry. These results support the supposition that the structure of photoexcited molecules in a **1B** crystal is similar to the ground-state structure of **1B**, while the structure of the photoexcited molecules in **1Y** crystals is similar to the ground-state structures of **1Y** and **1T$_{opt}$**.

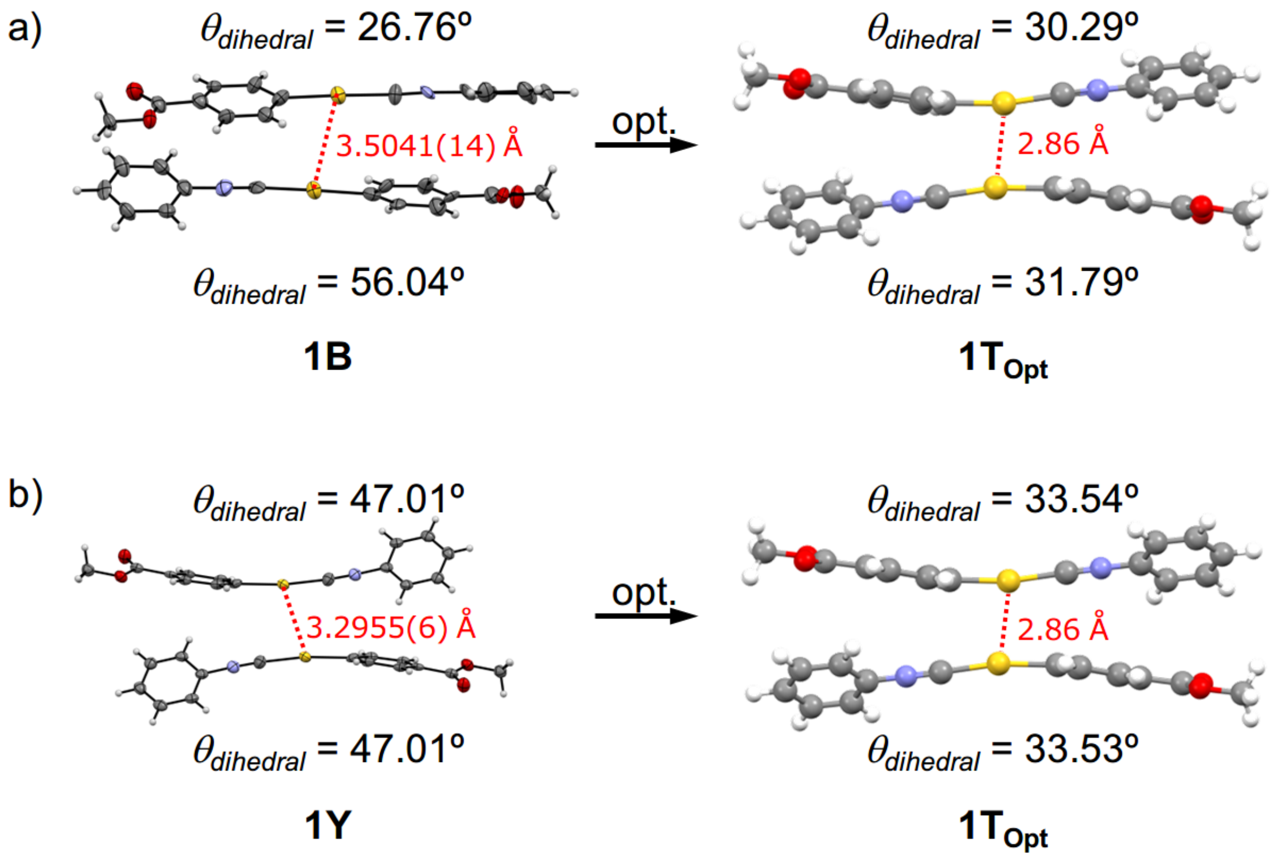

Fig. S13 Comparison of triplet state geometry optimization of **1B** a) and **1Y** b) to yield **1T$_{Opt}$** (PBEPBE/SDD).

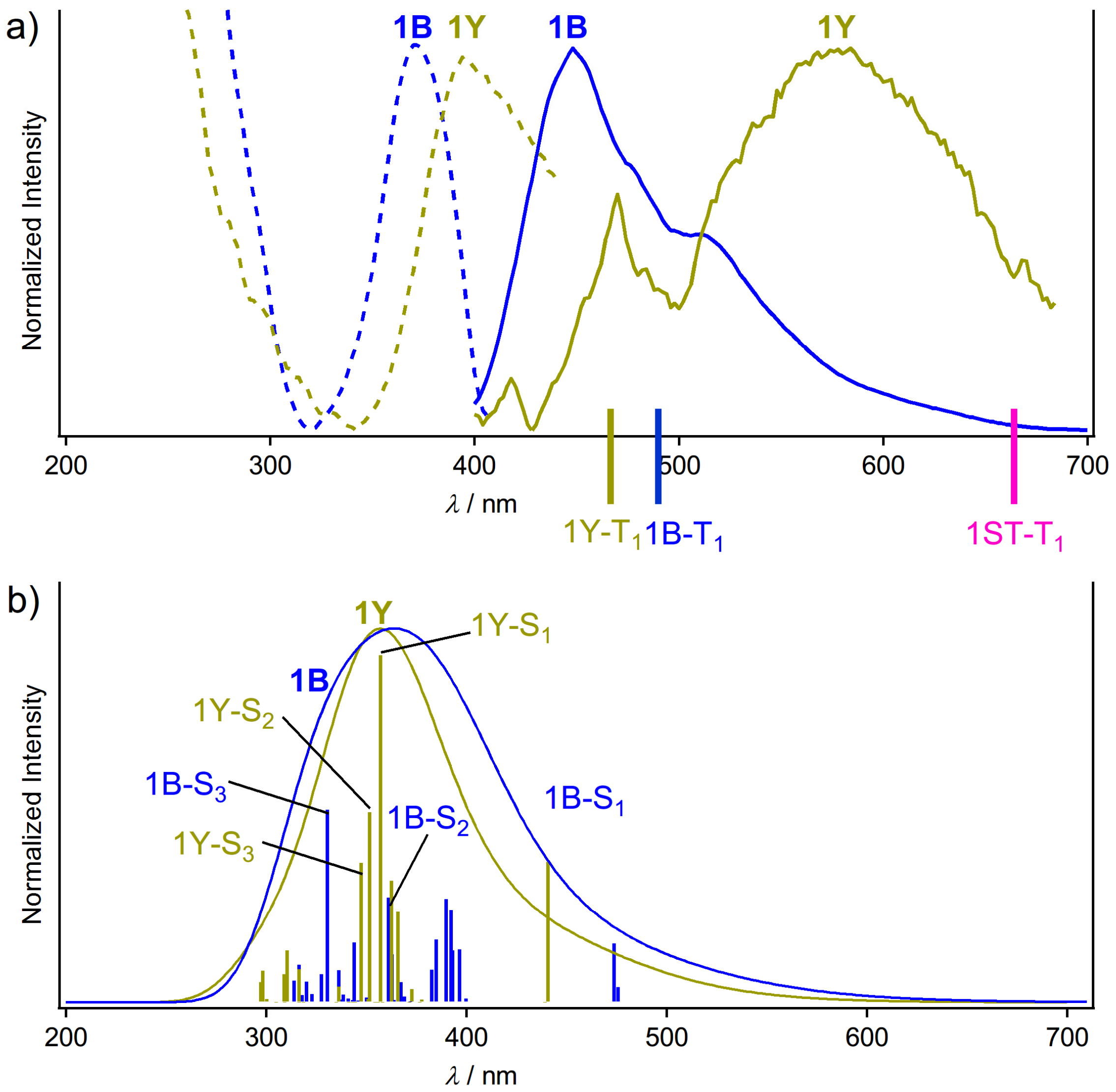

Fig. S14 Comparison of experimental and theoretical studies on optical properties of **1**. a) Blue lines: normalized excitation (dashed line, detected at 450 nm) and emission (solid line, λ_{ex} = 370 nm) spectra of **1B**. Greenish yellow lines: normalized excitation (dashed line, detected at 590 nm) and emission (solid line, λ_{ex} = 390 nm) spectra of **1Y**. b) Simulated UV/vis absorption spectra of **1B** (blue line) and **1Y** (greenish yellow line) based on the TDDFT calculations of the dimers derived from the corresponding single crystalline structures (PBEPBE/SDD).

Table S5. Selected singlet to singlet transitions for **1B** (PBEPBE/SDD).

states	calcd. energy	λ / nm	f /-	orbital transition	CI coefficients
**1B**					
1B-S$_1$	3.1759 eV	389.95	0.0443	HOMO−3 → LUMO	0.21442
				HOMO−2 → LUMO	−0.20367
				HOMO−2 → LUMO+1	0.55514
				HOMO−1 → LUMO+1	−0.15219
				HOMO → LUMO+1	0.12529
1B-S$_2$	3.3751 eV	361.17	0.0448	HOMO−5 → LUMO	0.10823
				HOMO−5 → LUMO+1	0.46106
				HOMO−4 → LUMO	0.21173
				HOMO−4 → LUMO+1	−0.44511
1B-S$_3$	3.7507 eV	330.56	0.0825	HOMO−3 → LUMO+2	0.21537
				HOMO−2 → LUMO+2	0.54284
				HOMO−2 → LUMO+4	−0.13567
				HOMO → LUMO+7	−0.26339

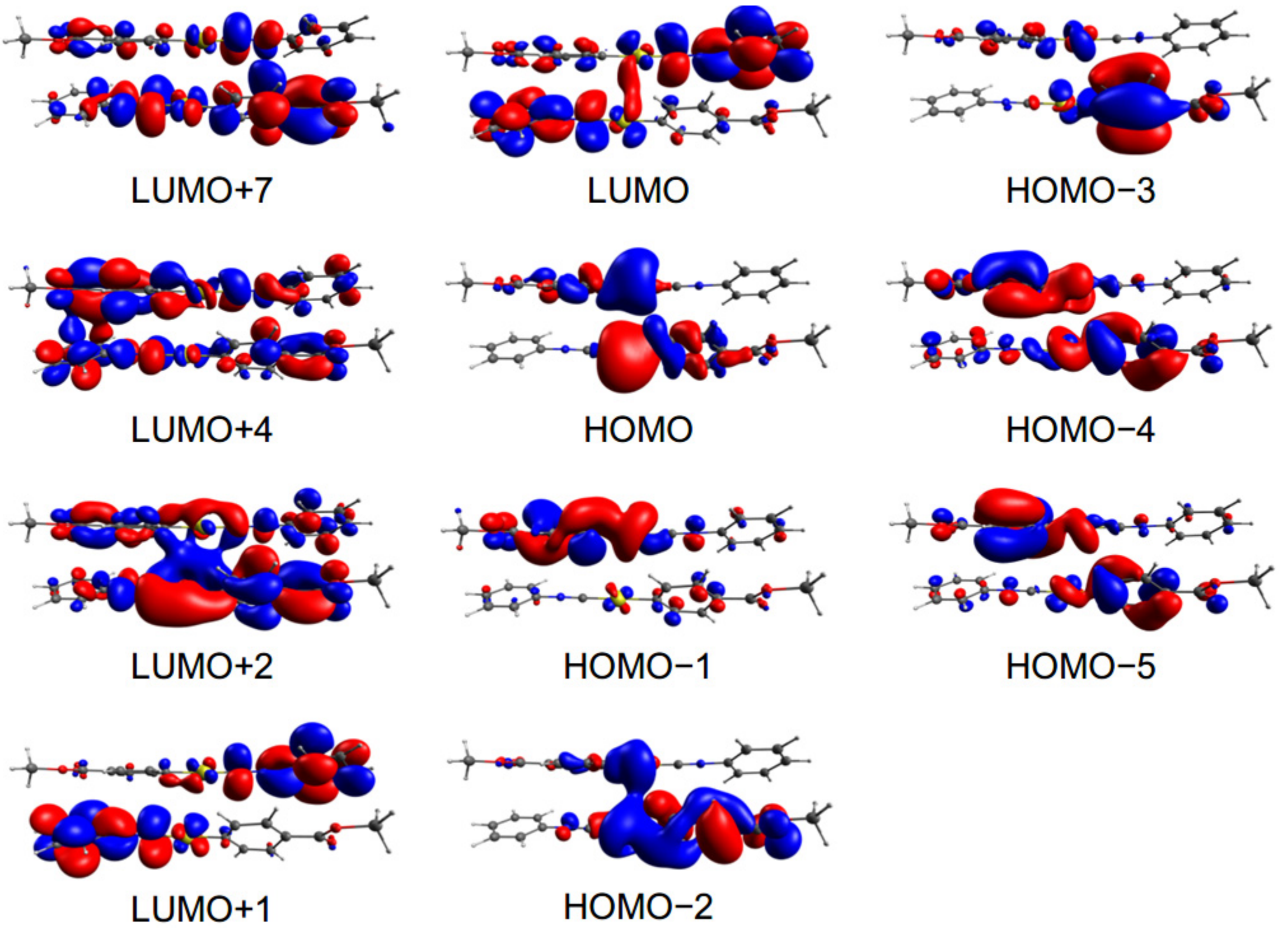

Table S6. Selected singlet to singlet transitions of **1Y** (PBEPBE/SDD).

states	calcd. energy	λ / nm	f /-	orbital transition	CI coefficients
**1Y**					
1Y-S_1	3.4295 eV	361.52	0.0813	HOMO−4 → LUMO	0.11097
				HOMO−3 → LUMO	0.37546
				HOMO−2 → LUMO+1	−0.23850
				HOMO → LUMO+2	0.51537
1Y-S_2	3.4725 eV	357.04	0.1468	HOMO−6 → LUMO	−0.12132
				HOMO−4 → LUMO	0.45782
				HOMO−3 → LUMO	−0.41193
				HOMO−2 → LUMO+1	−0.24006
				HOMO−1 → LUMO+1	−0.15460
1Y-S_3	3.5677 eV	347.52	0.0597	HOMO−6 → LUMO	0.66846
				HOMO−5 → LUMO+1	−0.13310
				HOMO−3 → LUMO	−0.12674

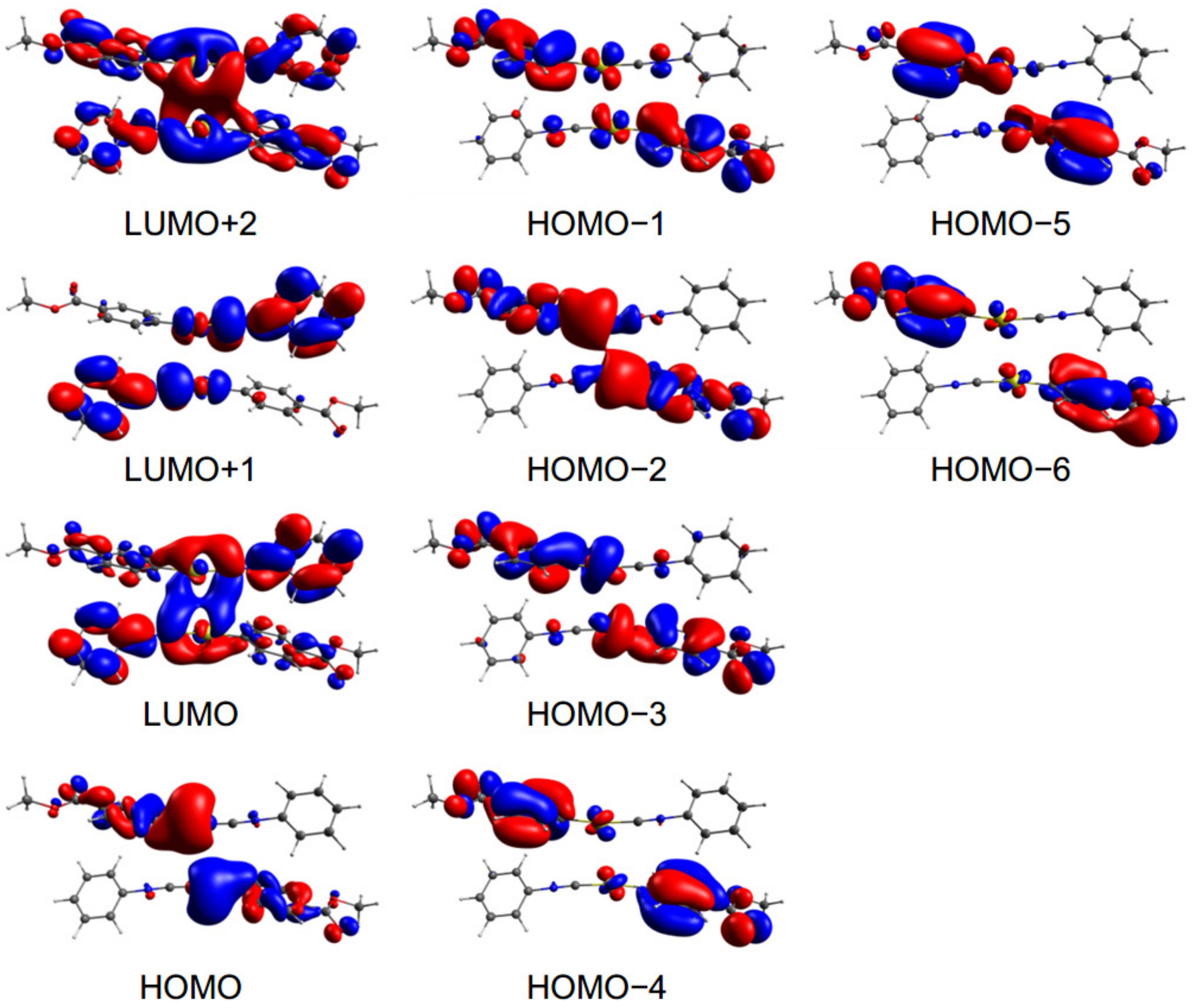

Table S7. Lowest singlet to triplet transitions for **1B** and **1Y** (PBEPBE/SDD).

states	calcd. energy	λ / nm	f /-	orbital transition	CI coefficients
**1B**					
1B-T_1	2.5243 eV	491.16	0.0000	HOMO → LUMO	0.70398

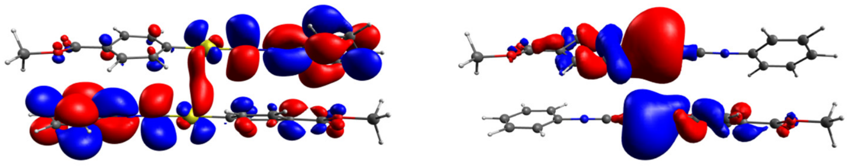

LUMO HOMO

states	calcd. energy	λ / nm	f /-	orbital transition	CI coefficients
**1Y**					
1B-T_1	2.6750 eV	463.49	0.0000	HOMO → LUMO	0.70529

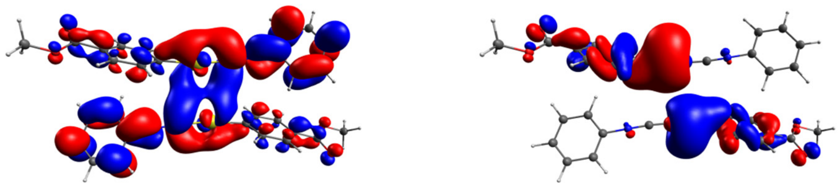

LUMO HOMO

states	calcd. energy	λ / nm	f /-	orbital transition	CI coefficients
**1ST$_{opt}$** (singlet model structure with **1T$_{opt}$** geometry)					
1ST-T_1	1.8661 eV	664.39	0.0000	HOMO → LUMO	0.70679

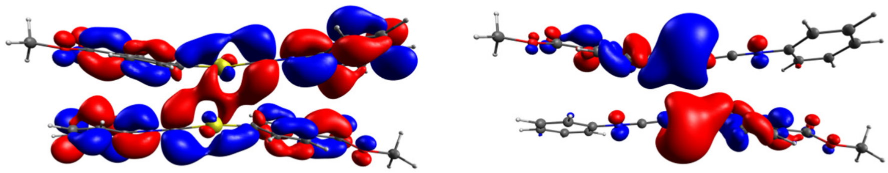

LUMO HOMO

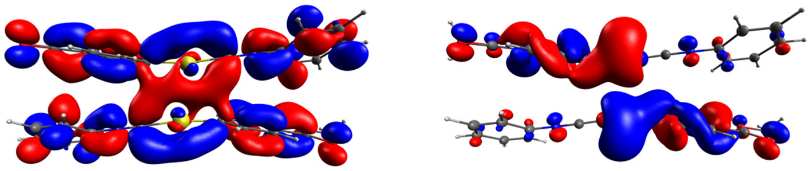

Fig. S15 SOMO of $\mathbf{1T_{Opt}}$ (PBEPBE/SDD).

11. Mechanical Response of 1B upon Strong Photoirradiation

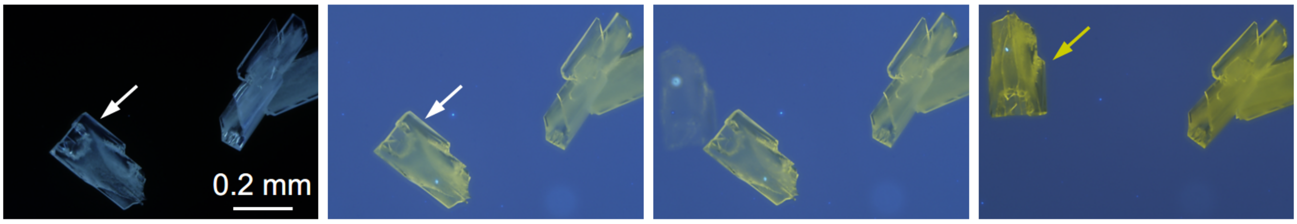

Fig. S16 A series of photographs of the photosalient effect of **1B** through the transformation into **1Y** under photoirradiation (367 nm, approx. 400 mW·cm^{-2}). Fourth picture was taken in 30 s after photoirradiation. White and yellow allows indicate the crystals before and after jump, respectively.

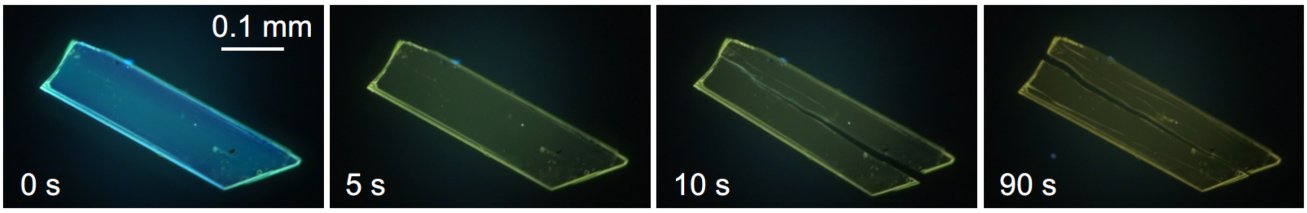

Fig. S17 A series of photographs of the crystal splitting of **1B** through the transformation into **1Y** under photoirradiation (367 nm, approx. 400 mW·cm^{-2}). These photographs were cropped from the Supplementary Movie S3.

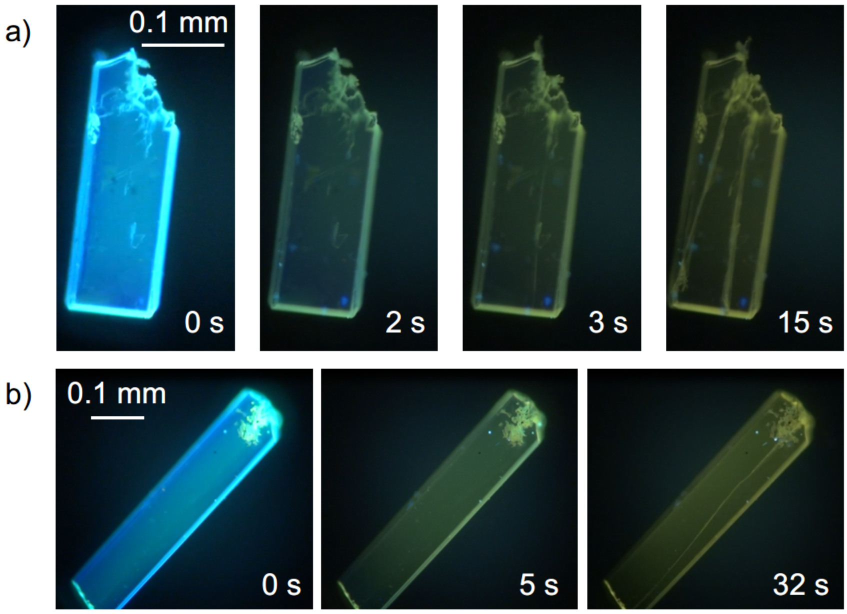

Fig. S18 A series of photographs of the crystal cracking of **1B** through the transformation into **1Y** under photoirradiation (367 nm, approx. 400 mW·cm^{-2}). These photographs were cropped from movies (data not shown).

12. References

1. A. Krasovskiy, V. Malakhov, A. Gavryushin, P. Knochel, *Angew. Chem. Int. Ed.* **2006**, *45*, 6040–6044.
2. Sheldrick, G. M. SHELXL-97, Program for the Refinement of Crystal Structures; University of Göttingen, Göttingen, Germany, **1997**.
3. Gaussian 09, Revision C.01, M. J. Frisch, G. W. Trucks, H. B. Schlegel, G. E. Scuseria, M. A. Robb, J. R. Cheeseman, G. Scalmani, V. Barone, B. Mennucci, G. A. Petersson, H. Nakatsuji, M. Caricato, X. Li, H. P. Hratchian, A. F. Izmaylov, J. Bloino, G. Zheng, J. L. Sonnenberg, M. Hada, M. Ehara, K. Toyota, R. Fukuda, J. Hasegawa, M. Ishida, T. Nakajima, Y. Honda, O. Kitao, H. Nakai, T. Vreven, J. A. Montgomery, Jr., J. E. Peralta, F. Ogliaro, M. Bearpark, J. J. Heyd, E. Brothers, K. N. Kudin, V. N. Staroverov, R. Kobayashi, J. Normand, K. Raghavachari, A. Rendell, J. C. Burant, S. S. Iyengar, J. Tomasi, M. Cossi, N. Rega, J. M. Millam, M. Klene, J. E. Knox, J. B. Cross, V. Bakken, C. Adamo, J. Jaramillo, R. Gomperts, R. E. Stratmann, O. Yazyev, A. J. Austin, R. Cammi, C. Pomelli, J. W. Ochterski, R. L. Martin, K. Morokuma, V. G. Zakrzewski, G. A. Voth, P. Salvador, J. J. Dannenberg, S. Dapprich, A. D. Daniels, Ö. Farkas, J. B. Foresman, J. V. Ortiz, J. Cioslowski, and D. J. Fox, Gaussian, Inc., Wallingford CT, 2009.
4. A. D. Becke, *Phys. Rev. A* **1988**, *38*, 3098–3100.
5. C. Lee, W. Yang, R. G. Parr, *Phys. Rev. B* **1988**, *37*, 785–789.
6. A. D. Becke, *J. Chem. Phys.* **1993**, *98*, 5648–5652.
7. T. H. Dunning, *J. Chem. Phys.* **1989**, *90*, 1007–1023.
8. Spartan ’10; Wavefunction, Inc.: Irvine, CA.
9. Avogadro: http://sourceforge.net/projects/avogadro/
